# Galectin 3 protects from cisplatin-induced acute kidney injury by promoting TLR-2-dependent activation of IDO1/Kynurenine pathway in renal DCs

**DOI:** 10.7150/thno.33959

**Published:** 2019-08-14

**Authors:** Vladislav Volarevic, Bojana Simovic Markovic, Marina Gazdic Jankovic, Bojana Djokovic, Nemanja Jovicic, C. Randall Harrell, Crissy Fellabaum, Valentin Djonov, Nebojsa Arsenijevic, Miodrag L. Lukic

**Affiliations:** 1 Department of Microbiology and Immunology, Center for Molecular Medicine and Stem Cell Research, Faculty of Medical Sciences, University of Kragujevac, 69 Svetozar Markovic Street, Kragujevac, Serbia; 2 Department of Genetics, Faculty of Medical Sciences, University of Kragujevac, 69 Svetozar Markovic Street, Kragujevac, Serbia; 3 Department of Histology and Embryology, Faculty of Medical Sciences, University of Kragujevac, 69 Svetozar Markovic Street, Kragujevac, Serbia; 4 Regenerative Processing Plant, LLC, 34176 US Highway 19 N Palm Harbor, Palm Harbor, Florida, United States of America; 5 Institute of Anatomy, University of Bern, 2 Baltzerstrasse, Switzerland

**Keywords:** cisplatin-induced acute kidney injury, Galectin 3, renal dendritic cells, Toll-like receptor-2, Indoleamine 2, 3-dioxygenase-1

## Abstract

Strategies targeting cross-talk between immunosuppressive renal dendritic cells (DCs) and T regulatory cells (Tregs) may be effective in treating cisplatin (CDDP)-induced acute kidney injury (AKI). Galectin 3 (Gal-3), expressed on renal DCs, is known as a crucial regulator of immune response in the kidneys. In this study, we investigated the role of Gal-3 for DCs-mediated expansion of Tregs in the attenuation of CDDP-induced AKI.

**Methods**: AKI was induced in CDDP-treated wild type (WT) C57BL/6 and Gal-3 deficient (Gal-3^-/-^) mice. Biochemical, histological analysis, enzyme-linked immunosorbent assay (ELISA), immunohistochemistry, real-time PCR, magnetic cell sorting, flow cytometry and intracellular staining of renal-infiltrated immune cells were used to determine the differences between CDDP-treated WT and Gal-3^-/-^ mice. Newly synthesized selective inhibitor of Gal-3 (Davanat) was used for pharmacological inhibition of Gal-3. Recombinant Gal-3 was used to demonstrate the effects of exogenously administered soluble Gal-3 on AKI progression. Pam3CSK4 was used for activation of Toll-like receptor (TLR)-2 in DCs. Cyclophosphamide or anti-CD25 antibody were used for the depletion of Tregs. 1-Methyl Tryptophan (1-MT) was used for pharmacological inhibition of Indoleamine 2,3-dioxygenase-1 (IDO1) in TLR-2-primed DCs which were afterwards used in passive transfer experiments.

**Results**: CDDP-induced nephrotoxicity was significantly more aggravated in Gal-3^-/-^ mice. Significantly reduced number of immunosuppressive TLR-2 and IDO1-expressing renal DCs, lower serum levels of KYN, decreased presence of IL-10-producing Tregs and significantly higher number of inflammatory IFN-γ and IL-17-producing neutrophils, Th1 and Th17 cells were observed in the CDDP-injured kidneys of Gal-3^-/-^ mice. Pharmacological inhibitor of Gal-3 aggravated CDDP-induced AKI in WT animals while recombinant Gal-3 attenuated renal injury and inflammation in CDDP-treated Gal-3^-/-^ mice. CDDP-induced apoptosis, driven by Bax and caspase-3, was aggravated in Gal-3^-/-^ animals and in WT mice that received Gal-3 inhibitor (CDDP+Davanat-treated mice). Recombinant Gal-3 managed to completely attenuate CDDP-induced apoptosis in CDDP-injured kidneys of Gal-3^-/-^ mice. Genetic deletion as well as pharmacological inhibition of Gal-3 in renal DCs remarkably reduced TLR-2-dependent activation of IDO1/KYN pathway in these cells diminishing their capacity to prevent transdifferentiation of Tregs in inflammatory Th1 and Th17 cells. Additionally, Tregs generated by Gal-3 deficient DCs were not able to suppress production of IFN-γ and IL-17 in activated neutrophils. TLR-2-primed DCs significantly enhanced capacity of Tregs for attenuation of CDDP-induced AKI and inflammation and expression of Gal-3 on TLR-2-primed DCs was crucially important for their capacity to enhance nephroprotective and immunosuppressive properties of Tregs. Adoptive transfer of TLR-2-primed WTDCs significantly expanded Tregs in the kidneys of CDDP-treated WT and Gal-3^-/-^ recipients resulting in the suppression of IFN-γ and IL-17-driven inflammation and alleviation of AKI. Importantly, this phenomenon was not observed in CDDP-treated WT and Gal-3^-/-^ recipients of TLR-2-primed Gal-3^-/-^DCs. Gal-3-dependent nephroprotective and immunosuppressive effects of renal DCs was due to the IDO1-induced expansion of renal Tregs since either inhibition of IDO1 activity in TLR-2-primed DCs or depletion of Tregs completely diminished DCs-mediated attenuation of CDDP-induced AKI.

**Conclusions**: Gal-3 protects from CDDP-induced AKI by promoting TLR-2-dependent activation of IDO1/KYN pathway in renal DCs resulting in increased expansion of immunosuppressive Tregs in injured kidneys. Activation of Gal-3:TLR-2:IDO1 pathway in renal DCs should be further explored as new therapeutic approach for DC-based immunosuppression of inflammatory renal diseases.

## Introduction

Cisplatin (cis-diamminedichloroplatinum II, CDDP) is one of the most effective chemotherapeutic agents 1. However, its clinical use is limited due to the severe side effects, including acute kidney injury (AKI) which has been observed in 30-40% of CDDP-treated patients 1. During glomerular filtration and tubular secretion, CDDP accumulates in the proximal tubular epithelial cells (PTECs) which absorb molecules from primary urine and are mainly exposed to urinary excreted CDDP 1. CDDP induces extensive production of free radicals and reactive oxygen species (ROS) in PTECs resulting in the development of oxidative stress which accelerates formation of advanced glycosylation end products (AGEs) and exacerbates AKI 1. Additionally, damage-associated molecular patterns (DAMPs), released from CDDP-injured PTECs, activate toll-like receptor (TLR)-4 on renal macrophages resulting in the production of inflammatory chemokines and cytokines which enable massive recruitment of circulating interferon gamma (IFN-γ) and interleukin (IL)-17-producing neutrophils and T cells in injured kidneys leading to the aggravation of AKI 2. In contrast to TLR-4, TLR-2 protects against CDDP-caused nephrotoxicity 3. Activation of TLR-2 promotes generation of tolerogenic and immunosuppressive phenotype in renal dendritic cells (DCs), which are considered as the main renal residential immune cells with nephroprotective function 4. Through the production of anti-inflammatory IL-10, renal DCs suppress detrimental IFN-γ and IL-17-driven immune response in injured kidneys and attenuate CDDP-induced inflammation 5-6. Nevertheless, inhibition of IL-10 production only partially reduces nephroprotective effects of renal DCs 6, suggesting that other DC-derived immunosuppressive mediators also contribute to the DC-dependent attenuation of CDDP-induced AKI.

Upon activation, tissue resident tolerogenic DCs express Indoleamine 2,3-dioxygenase 1 (IDO1) which metabolizes tryptophan to Kynurenine (KYN) 7. DC-derived KYN promotes expansion of Tregs in inflamed colon, lungs, brain, synovia, myocardium, skin and liver resulting in attenuated inflammation and enhanced regeneration of injured tissues 8. T regulatory cells (Tregs) are immunosuppressive cells which, 6 hours after CDDP-induced damage of PTECs, migrate in injured kidneys and in juxtacrine and paracrine manner, suppress IFN-γ and IL-17-producing neutrophils and T cells resulting in alleviation of AKI 9-10. Although DC-derived IDO1 represents possible molecular target for attenuation of CDDP-induced AKI, molecular mechanism which regulates IDO1-mediated cross-talk between renal DCs and Tregs in CDDP-injured kidneys is still unknown.

Considerable interest has recently arisen in the intriguing immunomodulatory properties of Galectin 3 (Gal-3), a member of β-galactoside-binding lectins, which regulates numerous biological processes in the kidneys including migration, proliferation and activation of resident and renal-infiltrated immune cells 11. A large number of studies reported different (pro-or anti-inflammatory) role of Gal-3 in the development and progression of organ specific inflammatory diseases 12-17. Interestingly, use of different disease-causing agents may induce opposite Gal-3-dependent effects in the same tissue 18-20, suggesting that Gal-3-dependent effects are determined by etiological agents and inflammatory microenvironment of injured tissue. In persistent or repetitive kidney injury, overexpression of Gal-3 promotes apoptosis and collagen I synthesis in renal cells enhancing transition of acute to chronic inflammation and fibrosis 11. Accordingly, elevated plasma levels of Gal-3 have been observed in patients with chronic kidney diseases and inhibition of Gal-3 prevented development of renal fibrosis 21-25. Oppositely, Galectin-3 has an important nephroprotective role in response to ischemic and nephrotoxic AKI 26-27. Gal-3 serves as a receptor for AGEs in the kidneys and is crucially important for protection towards AGE-dependent renal injury 28. Accordingly, genetic deletion of Gal-3 impairs uptake and removal of AGEs resulting in aggravation of AGE-induced glomerular injury and diabetic nephropathy 27. Having in mind that Gal-3 is constitutively expressed on renal DCs and that increased levels of AGEs, induced by CDDP, promote generation of tolerogenic phenotype in renal DCs 29, we analyzed the role of Gal-3 for DC-dependent alleviation of CDDP-induced AKI. By using complementary *in vitro* and *in vivo* approaches we demonstrated that genetic deletion as well as pharmacological inhibition of Gal-3 significantly impaired capacity of TLR-2-primed renal DCs to express IDO1 and produce immunosuppressive KYN which resulted in significantly reduced presence of renal-infiltrated Tregs and notably aggravated CDDP-induced AKI. Therefore, we propose that Gal-3 protects from CDDP-caused nephrotoxicity by promoting TLR-2-dependent activation of IDO1/KYN pathway in renal DCs resulting in increased expansion of immunosuppressive Tregs in injured kidneys.

## Material and Methods

**Animals.** Male, 6-8-week-old wild type (WT) and Gal-3^-/-^ C57BL/6 mice were used for the induction of CDDP-induced AKI. Breeding pairs of Gal-3^-/-^ and WT C57BL/6 mice of the same substrain were initially obtained from Dr. Daniel Hsu (University of California, Davis, USA) 30 and maintained in animal facilities of the Faculty of Medical Sciences, University of Kragujevac, Serbia. All animals received humane care and all experiments were approved by and conducted in accordance with, the Guidelines of the Animal Ethics Committee of the Faculty of Medical Sciences, University of Kragujevac, Serbia. Mice were housed in a temperature-controlled environment with a 12-h light-dark cycle and were administered standard laboratory chow and water *ad libitum*.

**Induction of AKI*.*** To generate CDDP-induced AKI, WT and Gal-3^-/-^ mice were injected with a single, intraperitoneal (i.p) dose of CDDP (16 mg/kg body weight). After mouse euthanasia (72 h after CDDP treatment), both kidneys were excised and blood samples were drawn from the inferior vena cava, as previously described 31.

**Administration of recombinant Gal-3 (rGal-3).** In order to evaluate the effects of rGal-3 in attenuation of CDDP-induced AKI, Gal-3^-/-^ mice received single intravenous injection of rGal-3 (5 μg; Peprotech, Rocky Hill, NJ, United States), 24 h before CDDP administration 20. Gal-3^-/-^ animals from control group received only saline.

**Pharmacological inhibition of Gal-3**. Gal-3 inhibitor (Davanat; kindly provided by Professor Klyosov and Professor Traber from Galectin Therapeutics Inc., Newton, MA) was intraperitoneally injected in CDDP-treated WT animals (100 μg/day), for three consecutive days before CDDP administration 17. WT animals from control group received only saline.

**Evaluation of CDDP-induced AKI.** CDDP-induced AKI was evaluated by biochemical and histological analysis, as previously described 31.

**Biochemical analysis.** Serum levels of urea and creatinine were determined to assess the renal function. After blood collection, serum levels of these toxicity markers were measured immediately using assay kits and blood chemistry analyzer, according to the manufacturer's instructions 31.

**Histopathological Analysis.** Kidney tissue was fixed in 10% buffered formalin, embedded in paraffin and cut at 5 μm thickness. Sections were stained with Hematoxylin and Eosin (H&E) and examined in a blinded manner. Histological sections were scored using a semi-quantitative scale designed to assess AKI-associated tubular injury (tubular epithelial cell loss, necrosis, tubular epithelial simplification, intratubular debris, and casts) by a pathologist blinded to the experimental groups (using >5 random fields/section, 4-5 mice/group). Tubule injury scores (ranging between 0 and 4) were based on the percentage of tubules affected as follows: 0 ≤ 10%, 1 = 10-25%, 2 = 26-50%, 3 = 51-75%, and 4 ≥ 75%, as previously described 31.

**Immunohistochemistry.** In order to assess expression of AGEs, Galectin-3 and TLR2 in the kidneys of WT and Gal-3^-/-^ mice, immunohistochemical (IHC) analysis was performed. Formalin-Fixed Paraffin-Embedded (FFPE) kidney tissue sections were incubated with rabbit anti-mouse AGE antibody (ab23722, Abcam Inc., Cambridge, MA, USA), rabbit anti-mouse Galectin 3 antibody (ab2785, Abcam Inc., Cambridge, MA, USA) and rabbit anti-mouse TLR2 antibody (ab213676, Abcam Inc., Cambridge, MA, USA) overnight at room temperature. Immunoreactivity was visualized by rabbit-specific conjugate (Expose Rb-Specific HRP/DAB Detection IHC Kit; Abcam) according to the manufacturer's instructions. Sections were photomicrographed with a digital camera mounted on light microscope (Olympus BX51), digitized, and analyzed Analysis was performed on 10 fields per section at 40x magnification. Results are presented as mean count of positive stained cells per field 16.

**Isolation of Renal-Infiltrated Immune Cells.** Isolation of immune cells from the kidneys was conducted as previously described 31. Briefly, the kidneys were cut into small pieces (1-2 mm in dimension) using a regular metal shaping blade and placed into the collagenase solution for 30-45 min in the incubator at 37 °C. The cells were filtered through a 70 μm nylon cell strainer and then, cells were pelleted by centrifuging 10 min at 400×g, at 4 °C. Pellet was resuspended in 4 mL of 40% Percoll solution and gently overlaid onto 4 mL of 80% Percoll solution. Slight whitish translucent layers of cells were collected from the interface of the two Percoll phases after centrifugation at 1500×g for 30 min. These cells were then collected and pelleted by centrifuging 10 min at 400×g, at 4 °C. Cells were resuspended in 1 mL of Dulbecco's Modified Eagle Medium (DMEM), and the total number of cells was determined by using trypan blue exclusion on a hemocytometer 31.

**Flow cytometry of renal-infiltrated immune cells.** For flow cytometry, 1×10^6^ of renal-infiltrated cells were incubated with anti-mouse CD45, Gr-1, F4/80, CD4, CD11c, CD80, CD86, MHC-II, TLR-2, Gal-3 monoclonal antibodies conjugated with fluorescein isothiocyanate (FITC), phycoerythrin (PE), peridinin chlorophyll protein (PerCP), or allophycocyanin (APC) (all from BD Biosciences, San Jose, CA, USA) following the manufacturer's instructions. Immune cells derived from the kidneys were concomitantly stained for the intracellular content of IFN-γ, IL-17, IL-4, IL-10, IL-12, T-bet, RORγt, STAT3, IDO1, IL-23 and forkhead box P3 (FoxP3) by using the fixation/permeabilization kit and anti-mouse monoclonal antibodies conjugated with fluorescein isothiocyanate (FITC), phycoerythrin (PE), peridinin chlorophyll protein (PerCP), and allophycocyanin (APC) (BD Bioscience). For intracellular cytokine staining, cells were stimulated with 50 ng/mL Phorbol 12-myristate-13-acetate (PMA) and 500 ng/mL Ionomycin for 5 h, and GolgiStop (BD Biosciences) was added. Cells were fixed in Cytofix/Cytoperm, permeated with 0.1% saponin, and stained with fluorescent Abs. Flow cytometry was conducted on a BD Biosciences FACSCalibur and data were analyzed by Flowing Software analysis program.

**Isolation and TLR-2-priming of DCs.** DCs were isolated from the kidneys of healthy WT and Gal-3^-/-^ mice by magnetic cell sorting. Single-cell suspensions of renal-infiltrated mononuclear cells were labelled with CD11c MicroBeads (Miltenyi Biotec). The labelled cells were subsequently positively selected using MACS Column (Miltenyi Biotec) and MACS Separator (Miltenyi Biotec), as previously described 32. Isolated renal DCs (3×10^5^/ mL) were primed with TLR-2 agonist Pam3CSK4 (300 ng/mL) for 24 h. As a naive control, renal DCs were cultured in the absence of TLR-2 ligand for 24h 32.

**Pharmacological inhibition of Gal-3 in DCs.** For pharmacological inhibition of Gal-3, unprimed and TLR-2-primed renal DCs were cultured in the presence of selective Gal-3 inhibitor (Davanat, 0.015 mg/mL) for 24 h, according to previously published protocol 33.

**Pharmacological inhibition of IDO1 activity in DCs.** IDO1 was inhibited in TLR-primed renal DCs (WTDCs^Pam3CSK4+1-MT^) and Gal-3^-/-^DCs (Gal-3^-/-^DCs^Pam3CSK4+1-MT^) by using 1-methyl tryptophan (1-MT; 2 mM; Sigma-Aldrich, Munich, Germany), as previously described 34.

**Transfer of TLR-2 primed DCs in CDDP-treated animals.** For transfer-based experiments, TLR-2-primed DCs, isolated from the kidneys of untreated WT and Gal‐3^-/-^ mice (WTDCs^Pam3CSK4^ and Gal‐3^-/-^DCs^Pam3CSK4^), were intravenously injected (5×10^5^cells/mouse) in CDDP-treated WT recipients (WT^WTDCsPam3CSK4^ and WT^Gal‐3-/-DCsPam3CSK4^) or Gal‐3^-/-^ recipients (Gal‐3^-/-WTDCsPam3CSK4^ and Gal‐3^-/-Gal‐3-/-DCsPam3CSK4^) two days prior CDDP administration 35. The extent of renal injury was evaluated as described above.

**Measurement of cytokines.** The commercial enzyme-linked immunosorbent assay (ELISA) sets (R&D Systems, Minneapolis, MN) were used to determine the concentration of IL-12, IL-1β, IL-6, IL-23, IL-17, IFN-γ and IL-10 in serum samples of saline and CDDP-treated animals, according to the manufacturer's instructions 31.

**Determination of IDO1 activity.** IDO1 activity in serums samples of saline and CDDP-treated mice as well as in supernatants of unprimed and TLR-2-primed WTDCs, Gal-3^-/-^DCs and WTDCs^Davanat^ was determined by spectrophotometric measuring of KYN 32.

**Isolation of neutrophils.** Neutrophils were isolated from healthy WT animals by magnetic cell sorting (Neutrophil Biotin-Antibody Cocktail, Miltenyi Biotec, Bergisch Gladbach, Germany), according to manufacturer's instructions. Isolated neutrophils were stimulated by lipopolysaccharide (LPS, 100 ng/mL) for 18 h and used in the co-culture experiments with Tregs 36.

**Isolation of Tregs.** Tregs were isolated from the population of renal-infiltrated mononuclear cells by magnetic cell sorting (Tregs isolation kit, Miltenyi Biotec, Bergisch Gladbach, Germany), according to manufacturer's instructions 37.

**Contact-independent co-culture of TLR-2-primed DCs and Tregs**. Tregs were co-cultured in the presence of WT DCs^Pam3CSK4^, WTDC^Pam3CSK4+Davanat^ or Gal-3^-/-^DCs^Pam3CSK4^, as previously described 38. Tregs and DCs were cultured physically separated using a 0.4 µm porous transwell system (Corning Incorporated, Life Sciences, France). Tregs were placed in the lower chamber of 24 well plate and cultured in the presence of WTDCs^Pam3CSK4^, WTDC^Pam3CSK4+Davanat^ or Gal-3^-/-^DCs^Pam3CSK^ which were seeded in the transwell inserts, and ratio between DCs and Tregs was 1:10.

After 48h of culture, non-primed Tregs or Tregs primed with WTDCs^Pam3CSK4^, WTDC^Pam3CSK4+Davanat^ or Gal-3^-/-^DCs^Pam3CSK^ were collected and used for co-culture experiments with neutrophils, ELISA assay, flow cytometry analysis and intracellular staining of FoxP3, IFN-γ, IL-17 and IL-10 or frozen at -80 °C until gene expression were measured by real-time PCR. Non-primed Tregs or Tregs primed with WTDCs^Pam3CSK4^, WTDC^Pam3CSK4+Davanat^ or Gal-3^-/-^DCs^Pam3CSK^ (2x10^6^ cells/ well) were collected, seeded at 24 well plate, cultured for additional 24h in fresh DMEM medium. Supernatants were collected and IFN-γ, IL-17 and IL-10 were determined by ELISA sets (R&D Systems, Minneapolis, MN), according to the manufacturer's instructions 31.

**Co-culture of Tregs and neutrophils.** Non-primed Tregs as well as Tregs, previously primed by WTDCs^Pam3CSK4^, WTDC^Pam3CSK4+Davanat^ or Gal-3^-/-^DCs^Pam3CSK4^, were co-cultured with LPS-activated neutrophils, as previously described 36. DC-primed Tregs and neutrophils were cultured physically separated using a 0.4 µm porous transwell system (Corning Incorporated, Life Sciences, France). Neutrophils were placed in the lower chamber of 24 well plate and cultured in the presence of non-primed Tregs or Tregs primed with WTDCs^Pam3CSK4^, WTDC^Pam3CSK4+Davanat^ or Gal-3^-/-^DCs^Pam3CSK^ which were seeded in the transwell inserts, and ratio between neutrophils and Tregs was 10:1. After 48 h, the expression of IFN-γ, IL-17 and IL-10 in neutrophils was evaluated by real time PCR analysis. Neutrophils were collected, seeded at 24 well plate (1x10^6^ cells/ well), cultured for additional 24 h in fresh DMEM medium. Supernatants were collected and IFN-γ, IL-17 and IL-10 were determined by ELISA sets (R&D Systems, Minneapolis, MN), according to the manufacturer's instructions 31.

**RNA isolation and real‐time PCR analysis**. Total RNA from kidneys, Tregs or neutrophils was extracted using TRIzol reagent (Invitrogen, Carlsbad, CA, USA) according to the manufacturer's instructions. Total RNA (2 μg) was reversely transcribed to cDNA using High-Capacity cDNA Reverse Transcription Kit (Applied Biosystems, Foster City, California, USA). qRT-PCR was performed using Power SYBR MasterMix (Applied Biosystems) and miRNA-specific primers for IFN-γ, IL-17, IL-10, FoxP3, T-bet, RORγT, Bax, Bcl-2, caspase-3 and β-actin as a housekeeping gene. qPCR reactions were initiated with a 10 min incubation time at 95 °C followed by 40 cycles of 95 °C for 15 s and 60 °C for 60 s in a Mastercycler ep realplex (Eppendorf, Hamburg, Germany). Relative expression of genes was calculated according to the formula 2-(Ct-Ctactin), where Ct is the cycle threshold of the gene of interest and Ctactin is the cycle threshold value of the housekeeping gene (β-actin) 38.

**Transfer of Tregs.** Tregs were fluorescence-labeled using pre-incubation with carboxyfluorescein diacetate succin-imidyl ester (CFSE; Molecular Probes, Thermo FisherScientific, Stockholm, Sweden) according to the manufacturer's instructions. For transfer experiments non-primed Tregs, Tregs primed with WTDC^Pam3CSK4^, WTDC^Pam3CSK4+Davanat^ or Gal-3^-/-^ DC^Pam3CSK4^ (1x10^6^ Tregs/ mouse) were intravenously injected in CDDP-treated animals, 18 h before induction of AKI.

**Depletion of Treg cells.** For the depletion of Tregs, CDDP-treated WT^WTDCPam3CSK4^ and Gal-3^-/-WTDCPam3CSK4^ mice received either cyclophosphamide (CY, Galenika A.D., Belgrade, Serbia) 3 days before CDDP administration, at a dose of 10 mg/kg which was reported to selectively deplete Tregs but not DCs 19 or anti-CD25 (P61) monoclonal antibody (250 μg per mouse; eBioscience; San Diego, CA, USA) 39.

**Isolation and transfer of macrophages in CDDP-treated mice.** Macrophages, isolated from healthy WT and Gal-3^-/-^ mice, were stimulated with Pam3CSK4 (300 ng/mL; WTMϕ^Pam3CSK4^ and Gal-3^-/-^Mϕ^Pam3CSK4^) and injected via the tail vein (1x10^6^/ mouse) 2 h before induction of AKI, as previously suggested 40.

**Statistics*.*** Data were expressed as the Mean ± standard error of the mean (SEM) for each group. Results were analyzed by Student's t test and Pearson's or Spearman's correlation coefficient. Statistical analyses were performed using SPSS 23.0 for Windows software (SPSS Inc., Chicago, IL). The difference was considered significant when p<0.05.

## Results

### Genetic deletion of Gal-3 significantly aggravates AKI and inflammation

**Figure 1:**
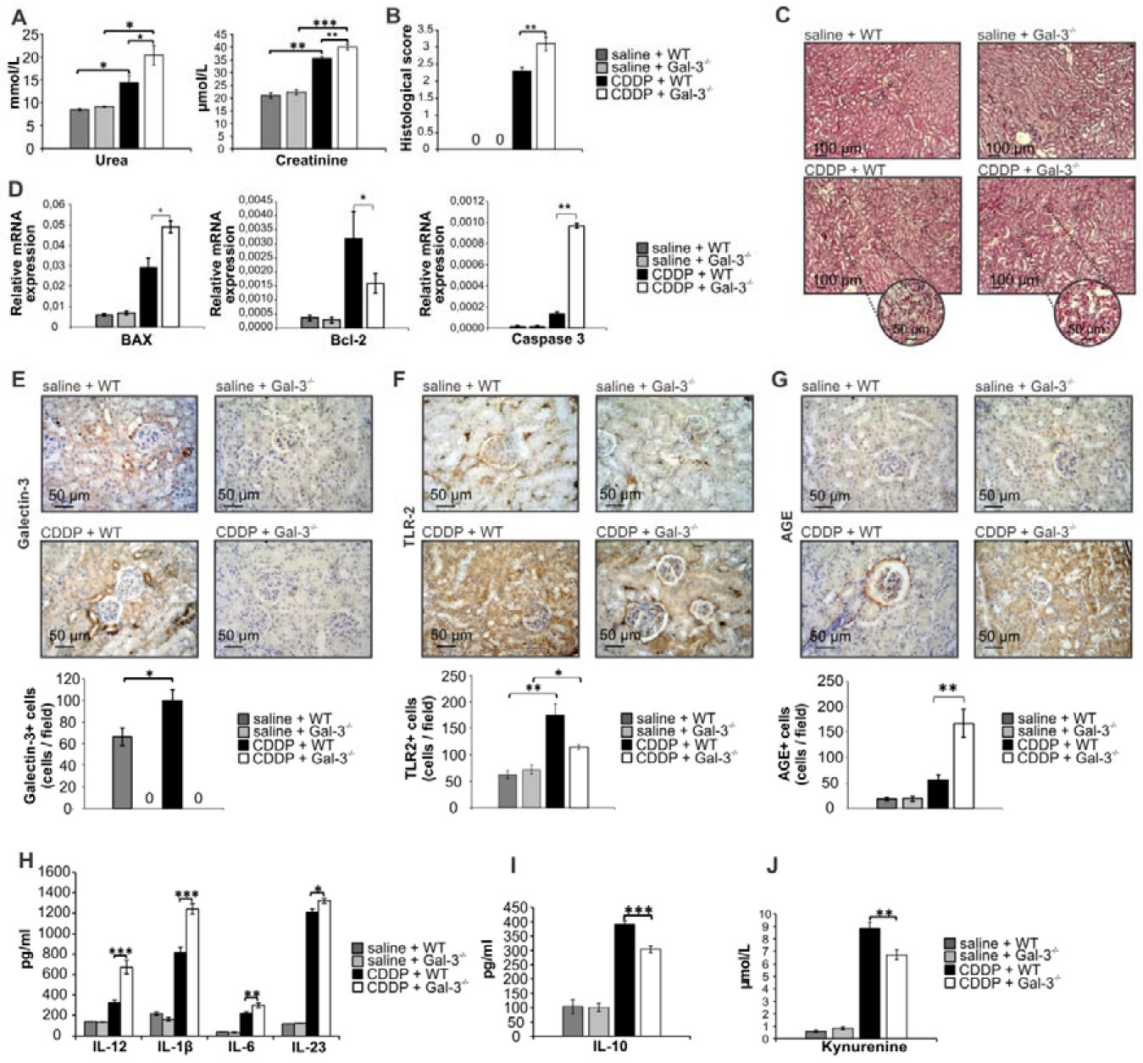
** Genetic deletion of Gal-3 significantly aggravates CDDP-induced AKI, apoptosis and inflammation.** WT and Gal-3^-/-^ mice were injected with a single, intraperitoneal dose of CDDP (16 mg/kg body weight). Acute renal failure was aggravated in Gal-3^-/-^ mice 72 h after CDDP treatment, as evidenced by increased concentration of urea and creatinine (A), higher histological score (B) and more severe tubular epithelial cell injury, tubular dilation, and intra-tubular cast formation in the cortex of CDDP-injured kidneys (C). Genetic deletion of Gal-3 significantly increased apoptosis in CDDP-injured kidneys as evidenced by increased expression of Bax and caspase-3 and decreased expression of Bcl-2 (D). CDDP treatment significantly up-regulated expression of Gal-3 (E) and TLR-2 (F). Gal-3 deletion significantly attenuated CDDP-induced increase of TLR-2 in the kidneys and significantly increased expression of AGEs (G). Significantly increased serum levels of Th1 and Th17-promoting inflammatory cytokines (IL-12, IL-1β, IL-6 and IL-23) (H) and decreased levels of immunosuppressive IL-10 (I) and KYN (J) were observed in CDDP-treated Gal-3^-/-^ animals. Data from two individual experiments with 8 mice per group are shown as Mean ± SEM; *p<0.05, **p<0.01,***p<0.001.

CDDP caused significant renal dysfunction in WT and Gal-3^-/-^ mice as reflected by a marked elevation of serum urea and creatinine (Figure 1A). Importantly, CDDP-induced nephrotoxicity was significantly more aggravated in Gal-3^-/-^ mice. Serum levels of urea (p<0.05, Figure 1A left panel) and creatinine (p<0.01, Fig.1A right panel) were significantly higher in CDDP-treated Gal-3^-/-^ mice when compared to similarly treated WT animals. An aggravated deterioration of kidney function, observed in CDDP-treated Gal-3^-/-^ mice, was confirmed by histological score (Figure 1B). Kidneys obtained from control animals had normal histology (Figure 1C, upper panels). CDDP treatment induced more severe tubular epithelial cell injury, tubular dilation, and intra-tubular cast formation in Gal-3^-/-^ mice compared to WT animals (Figure 1C, lower panels), which resulted in significantly higher histological score (p<0.01, Figure 1B) in CDDP-treated Gal-3^-/-^ mice, confirming that genetic deletion of Gal-3 remarkably aggravated AKI.

It is well known that CDDP induces apoptosis of proximal tubular epithelial cells 41 and that Gal-3 has anti-apoptotic function in epithelial cells 42. Accordingly, significantly higher expression of pro-apoptotic Bax and caspase 3 and significantly lower expression of anti-apoptotic Bcl-2 were noticed in the CDDP-injured kidneys of Gal-3^-/-^ mice compared to their WT counterparts (p<0.05 for Bax and Bcl-2; p<0.01 for caspase 3; Figure 1D).

TLR-2 is constitutively expressed in the kidneys and protects against CDDP-induced AKI by promoting autophagy in tubular epithelial cells 43. Since autophagy delays apoptosis of tubular epithelial cells 44, we analyzed whether Gal-3 deficiency affected TLR-2 expression in CDDP-injured kidneys. Immunohistochemical analysis revealed that expression of both Gal-3 (p<0.05; Figure 1E) and TLR-2 (p<0.01; Figure 1F) were significantly increased in the kidneys upon CDDP administration. Importantly, genetic deletion of Gal-3 significantly reduced expression of TLR-2 in CDDP-treated mice (p<0.05; Figure 1F), suggesting that Gal-3 has important role in TLR-2-dependent nephroprotection.

Additionally, Gal-3 deficiency resulted with reduced uptake and enhanced accumulation of AGEs in the injured kidneys of CDDP-treated Gal-3^-/-^ mice (p<0.01; Figure 1G) which corresponded to the aggravated renal injury (Fig.1A-C), apoptosis (Figure 1D) and inflammation (Figure 1H-J). Significantly higher concentrations of pro-inflammatory cytokines which are necessary for the induction of Th1 (IL-12 (p<0.001)) and Th17 immune response (IL-1β (p<0.001), IL-6 (p<0.01), IL-23 (p<0.05)) were found in serum samples of CDDP-treated Gal-3^-/-^ mice when compared to similarly treated WT mice (Figure 1H). Additionally, significantly lower serum levels of anti-inflammatory and immunosuppressive mediators (IL-10 (p<0.001, Figure 1I)) and kynurenine (KYN, p<0.01, Figure 1J) were observed in CDDP-treated Gal-3^-/-^ mice, suggesting that Gal-3 has important immuno-regulatory role in CDDP-induced kidney injury and inflammation.

### Pharmacological inhibitor of Gal-3 aggravated CDDP-induced AKI in WT animals while recombinant Gal-3 attenuated renal injury and inflammation in CDDP-treated Gal-3^-/-^ mice

**Figure 2:**
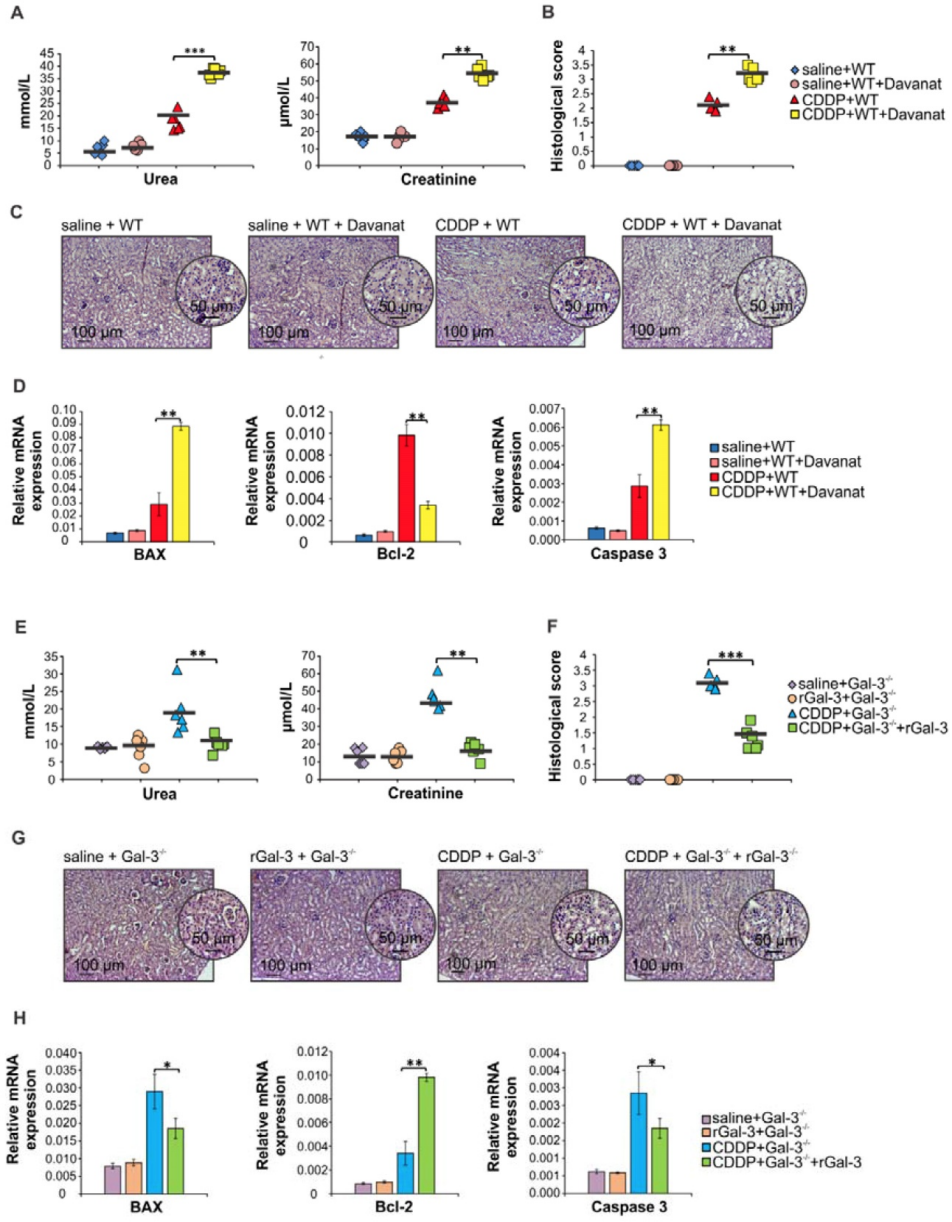
** Davanat significantly aggravated CDDP-induced AKI in WT animals while rGal-3 attenuated renal injury and inflammation in CDDP-treated Gal-3^-/-^ mice.** Davanat was intraperitoneally injected in CDDP-treated WT animals (100 μg/day), for three consecutive days before CDDP administration (16 mg/kg body weight). Gal-3^-/-^ mice received single intravenous injection of rGal-3 (5 μg), 24 hours before CDDP administration. Davanat significantly enhanced CDDP-induced AKI in WT mice, as evidenced by elevated serum levels of urea and creatinine (A) and significantly increased histological score (B). Histological analysis revealed more severe tubular epithelial cell injury in the cortex of the kidneys of CDDP+Davanat-treated WT mice (C). Expression of pro-apoptotic Bax and caspase-3 were significantly higher and expression of anti-apoptotic Bcl-2 was significantly lower in the kidneys of CDDP+Davanat-treated WT mice compared to CDDP-only-treated WT animals (D).Serum levels of urea and creatinine (E), histological score (F), injury of tubular epithelial cells (G) and expression of pro-apoptotic Bax and caspase-3 (H) were significantly lower, while expression of anti-apoptotic Bcl-2 was significantly higher (H) in the kidneys of CDDP+rGal-3-treated Gal-3^-/-^ mice. Individual data points with Mean, obtained in one experiment with 6 mice per group; *p<0.05, **p<0.01,***p<0.001.

In order to confirm protective role of Gal-3 in CDDP-caused AKI, we analyzed the effects of Gal-3 inhibitor (Davanat) and recombinant Gal-3 (rGal-3) on the development of CDDP-induced acute renal failure in WT and Gal-3^-/-^ animals. As it is shown in Figure S1, Davanat inhibited expression of Gal-3 in saline and CDDP-injured kidneys. Importantly, Davanat significantly enhanced CDDP-induced AKI in WT mice, as evidenced by elevated serum levels of urea and creatinine (p<0.001 for urea and p<0.01 for creatinine; Figure 2A) and significantly increased histological score (p<0.01; Figure 2B). Histological analysis revealed more severe tubular epithelial cell injury in the kidneys of CDDP+Davanat-treated WT mice (Figure 2C). Additionally, expression of pro-apoptotic Bax and caspase-3 were significantly higher and expression of anti-apoptotic Bcl-2 was significantly lower in the kidneys of CDDP+Davanat-treated WT mice compared to CDDP-only-treated WT animals (p<0.01; Figure 2D), confirming important anti-apoptotic role of Gal-3 in CDDP-induced AKI.

Complementary to these findings are results obtained after administration of rGal-3 in CDDP-treated Gal-3^-/-^ mice (Figure 2E-H). Serum levels of urea and creatinine (p<0.01; Figure 2E), histological score (p<0.001; Figure 2F), injury of tubular epithelial cells (Figure 2G) and expression of pro-apoptotic Bax and caspase-3 (p<0.05; Figure 2H) were significantly lower, while expression of anti-apoptotic Bcl-2 was significantly higher (p<0.01; Figure 2H) in the kidneys of CDDP-treated Gal-3^-/-^ mice that received rGal-3.

### Gal-3 deficiency affected phenotype and function of renal-infiltrated neutrophils and macrophages in CDDP-treated animals

**Figure 3:**
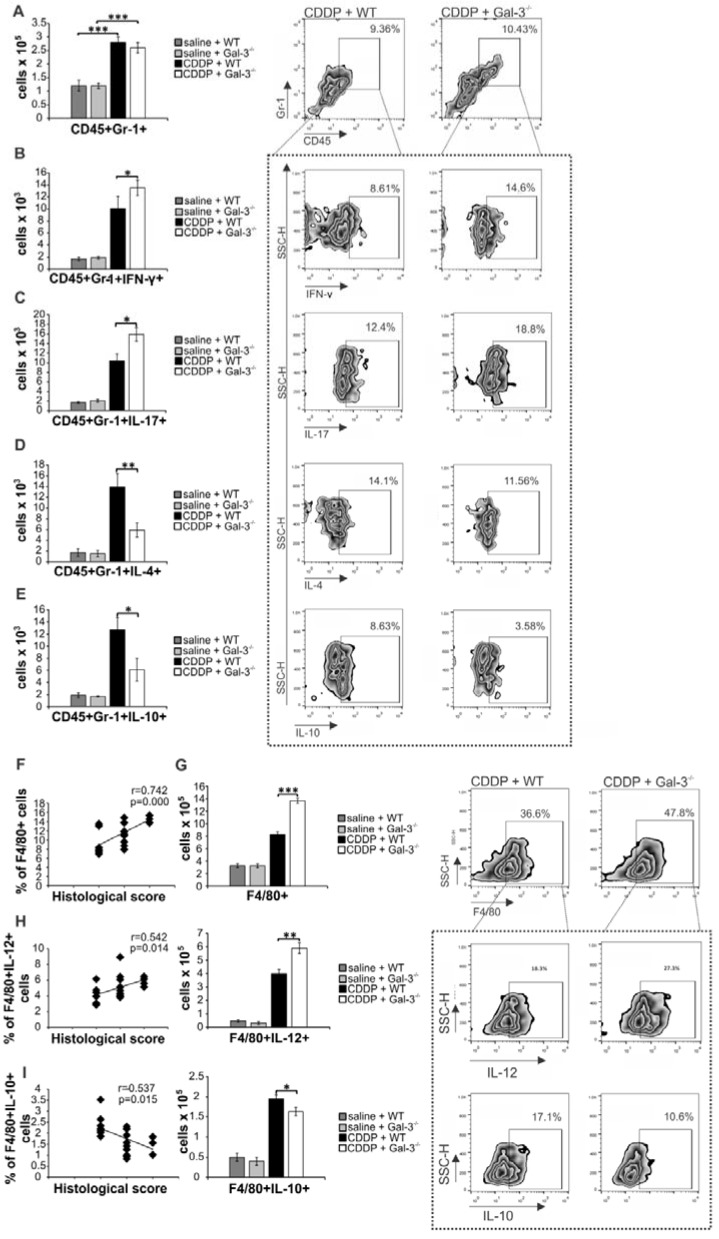
** Gal-3 deficiency promoted development of inflammatory phenotype in renal-infiltrated neutrophils and macrophages.** Bar graphs (left panels) and representative density plots obtained by flow cytometry analysis and intracellular staining of renal-infiltrated neutrophils and macrophages derived from saline or CDDP-treated WT and Gal-3^-/-^ mice, 72 h after CDDP administration (16 mg/kg body weight). There was no significant difference in total number of CD45+Gr-1+neutrophils (gated as CD45+Gr-1+cells in population of renal infiltrated cells) between CDDP-injured kidneys of WT and Gal-3^-/-^ animals (A). Significantly higher number of inflammatory, IFN-γ (B) and IL-17-producing neutrophils (C), and notably lower total number of IL-4-producing (D) and IL-10 producing neutrophils (E) were noticed in the kidneys of CDDP-treated Gal-3^-/-^ mice. Representative density plots show percentages of IFN-y-, IL-17-, IL-4- and IL-10-producing neutrophils (Fig.2B-E), gated in the population of CD45+Gr-1+ renal infiltrated cells. Strong positive correlation was observed between histological score and total number of renal-infiltrated macrophages (F) which was found in significantly higher number in the kidneys of CDDP-treated Gal-3^-/-^ mice (G). Representative density plots showing higher percentage of macrophages in renal infiltrated cells of CDDP-treated Gal-3^-/-^ mice compared to similarly treated WT animals (G, right panel). Macrophages are determined as F4/80+cells, gated in population of CD45+CD11c- renal infiltrated cells. Significantly higher number of IL-12-producing M1 macrophages (H, left panel), but lower number of IL-10-producing M2 macrophages (I, right panel) were noticed in the kidneys of CDDP-treated Gal-3^-/-^ mice. Positive correlation was observed between histological score and total number of IL-12-producing renal-infiltrated M1 macrophages (H, middle panel) and negative correlation was noticed between histological score and total number of IL-10-producing renal-infiltrated M2 macrophages (I, middle panel). Representative density plots show percentages of IL-12-producing (H, right panel) and IL-10-producing macrophages (I, right panel), gated in the population of F4/80+ renal infiltrated cells of CDDP-treated WT and Gal-3^-/-^ animals. Data from two individual experiments with 8 mice per group are shown as Mean ± SEM; *p<0.05, **p<0.01,***p<0.001.

An increase in serum concentration of inflammatory cytokines (IL-1β and IL-6) is associated with a massive influx of neutrophils in CDDP-injured kidneys 45. Since we noticed significantly higher serum levels of IL-1β and IL-6 in CDDP-treated Gal-3^-/-^ mice (Figure 1H), we compared phenotype and function of renal-infiltrated neutrophils between these two experimental groups (Figure 3A-E). CDDP induced massive influx of neutrophils in kidneys of WT and Gal-3^-/-^ mice without significant difference in their total number (Figure 3A). However, intracellular staining revealed that significantly higher number of inflammatory, IFN-γ and IL-17-producing neutrophils infiltrated the kidneys of CDDP-treated Gal-3^-/-^ mice (Figure 3B-C, p<0.05). Additionally, reduced presence of nephroprotective IL-4 (Figure 3D, p<0.01) and IL-10-producing neutrophils (Figure 3E, p<0.05) 46-48 were found in CDDP-injured kidneys of Gal-3^-/-^mice, suggesting that Gal-3 deficiency affected phenotype and function of renal-infiltrated neutrophils of CDDP-treated animals.

Gal-3 regulates secretion of cytokines and chemokines in macrophages playing important role in modulation of organ-specific and systemic inflammatory diseases 49. In line with previously published studies 50, 51, we noticed positive correlation between the extent of AKI and total number of renal-infiltrated macrophages (Figure 2F). Importantly, aggravated acute renal failure, observed in CDDP-injured Gal-3^-/-^ mice compared to similarly treated WT animals (Figure 1), corresponded to the notably higher presence of renal-infiltrated F4/80+ macrophages (Figure 3G, P<0.001).

It is well known that CDDP treatment induces enhanced expression of IL-12 in inflammatory M1 macrophages resulting in the progression of AKI and, at the same time, suppresses generation of alternatively activated and renoprotective, IL-10-producing M2 macrophages 52. In line with these findings, we noticed positive correlation between percentage of M1 macrophages and the extent of renal injury in CDDP-treated WT and Gal-3^-/-^ mice (Figure 3H, left panel) and negative correlation between percentage of renal-infiltrated M2 macrophages and histological score of CDDP-treated WT and Gal-3 deficient animals (Figure 3I, left panel). Importantly, significantly higher number of inflammatory, IL-12-producing macrophages (p<0.01; Figure 3H right panel) and remarkably reduced presence of nephroprotective, IL-10-producing M2 macrophages (p<0.05; Figure 3I right panel) in the kidneys of CDDP-treated Gal-3^-/-^ mice, suggesting that Gal-3 had important role in regulation of CDDP-induced polarization of renal macrophages.

Since Gal-3 needs association with TLR-2 for modulation of macrophage function 53, we investigated whether genetic deletion of Gal-3 alters capacity of TLR-2-primed macrophages to modulate CDDP-induced nephrotoxicity. Serum levels of urea and creatinine (Figure S2A) and histological score (Figure S2B) were not significantly different between CDDP-treated WT recipients of TLR-2-primed WT and Gal-3^-/-^macrophages, suggesting that increased infiltration of M1 macrophages and decreased presence of M2 macrophages in damaged kidneys of CDDP-treated Gal-3^-/-^ mice was just a reflection of the severity of AKI rather than its cause and that aggravated CDDP-induced AKI, seen in Gal-3^-/-^ animals, developed due to the dysfunction of other Gal-3-expressing regulatory immune cells with nephroprotective function.

### Gal-3 deficiency significantly increased total number of inflammatory Th1 and Th17 cells and remarkably reduced presence of regulatory T cells in CDDP-injured kidneys

**Figure 4:**
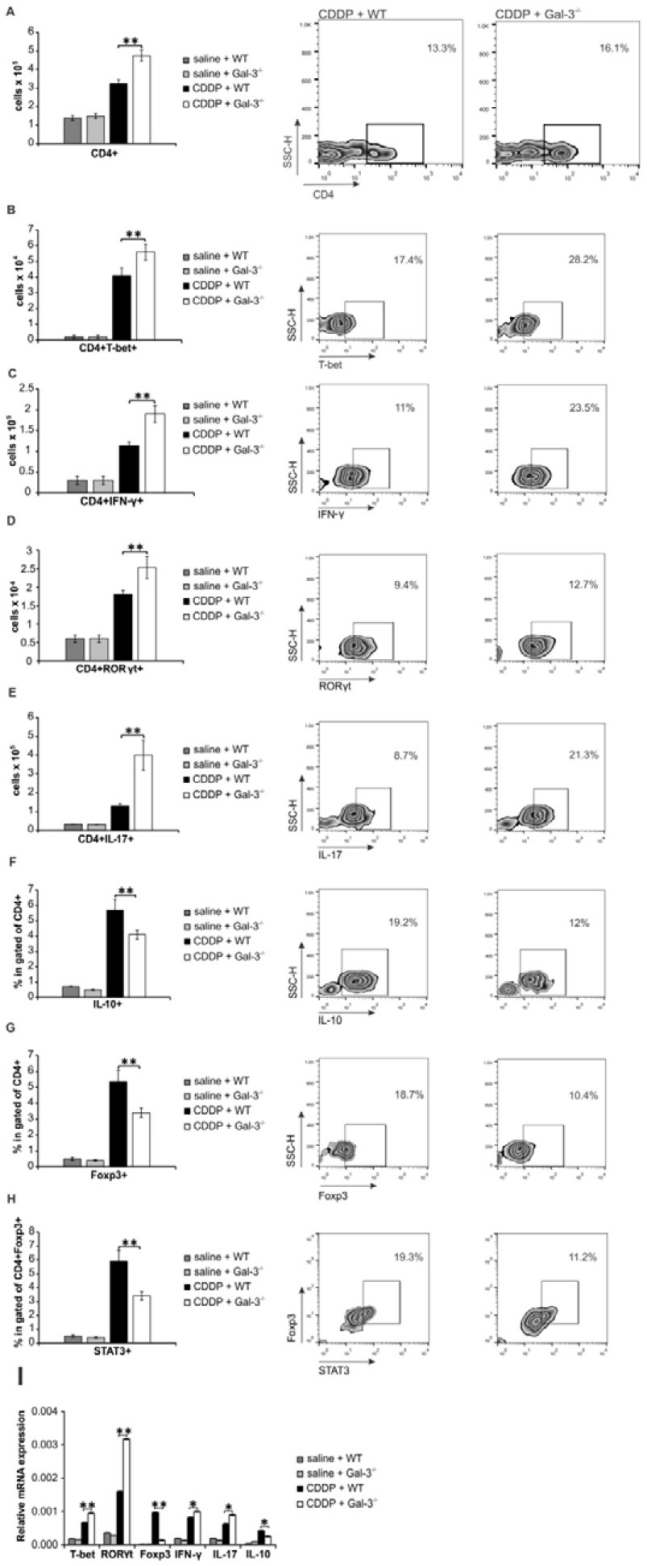
** Gal-3 deficiency significantly increased total number of inflammatory Th1 and Th17 cells and reduced presence of regulatory T cells in CDDP-injured kidneys.** Bar graphs (left panels) and representative density plots obtained by flow cytometry analysis and intracellular staining of renal-infiltrated CD4+T cells derived from saline or CDDP-treated WT and Gal-3^-/-^ mice, 72 h after CDDP administration (16 mg/kg body weight). Gal-3 deficiency resulted with increased presence of CD4+T cells in the CDDP-injured kidneys (A, left panel). Representative density plots showing CD4+ T lymphocytes, gated as CD4+ cells in population of renal infiltrated cells (A, right panel). Significantly higher total number of T-bet-expressing (B) and IFN-γ-producing (C) CD4+Th1 cells and RORγT-expressing (D) and IL-17-producing (E) CD4+Th17 cells, but reduced presence of immunosuppressive IL-10-producing CD4+T cells (F), CD4+FoxP3+ Tregs (G) and STAT-3-expressing Treg (H) were noticed in the kidneys of CDDP-treated Gal-3^-/-^ mice. Representative density plots showing percentages of T-bet-expressing, IFN-γ-producing, RORγT-expressing, IL-17-producing, IL-10-producing, FoxP3-expressing cells, gated in the population of CD4+Tcells (B-G, right panels) and percentage of STAT-3-expressing cells in gated population of CD4+FoxP3+ Tregs (H, right panel), isolated from the kidneys of CDDP-treated WT and Gal-3-/- mice, 72h after CDDP administration. Real-time PCR gene analysis showing significantly higher mRNA expression of T-bet, IFN-γ, RORγT, IL-17 and lower mRNA expression of FoxP3 in CDDP-injured kidneys of Gal-3-/- mice compared to similarly treated WT animals (I). Data from two individual experiments with 8 mice per group are shown as Mean ± SEM; *p<0.05, **p<0.01,***p<0.001.

Having in mind that crosstalk between renal-infiltrated neutrophils and Th17 cells as well as interaction between renal macrophages and Th1 cells have been implicated in the pathogenesis of AKI 54, 55, we analyzed phenotype and function of effector CD4+T cells in the CDDP-injured kidneys of WT and Gal-3^-/-^ mice (Figure 4A-H). As shown in Figure 4A, significantly higher number of CD4+T cells was present in the kidneys of CDDP-treated Gal-3^-/-^ mice (p<0.01). Intracellular staining revealed that notably higher number of inflammatory T-bet expressing and IFN-γ producing Th1 cells (Figure 4B-C, p<0.01) as well as RORγT expressing and IL-17-producing Th17 cells (Figure 4D-E, p<0.01) infiltrated injured kidneys of CDDP-treated Gal-3^-/-^ animals than their WT counterparts.

In line with these data were results obtained by real-time PCR gene expression analysis (Figure 4I). There was significantly higher mRNA expression of T-bet, IFN-γ, RORγT and IL-17 in the kidneys of CDDP-treated Gal-3^-/-^ mice compared to similarly treated WT animals (p<0.05 for IFN-γ, IL-17 and IL-10; p<0.01 for T-bet, RORγT; Figure 4I).

Additionally, Gal-3 deficiency significantly attenuated mRNA expression of FoxP3 and IL-10 (p<0.05 for IL-10; p<0.01 for FoxP3; Figure 4I) and remarkably reduced presence of renoprotective and immunosuppressive IL-10-producing, FoxP3 and STAT3-expressing regulatory T cells in CDDP-injured kidneys (Figure 4F-H, p<0.01) 56, indicating the important role of Gal-3 for generation of Tregs in CDDP-induced AKI.

**Figure 5:**
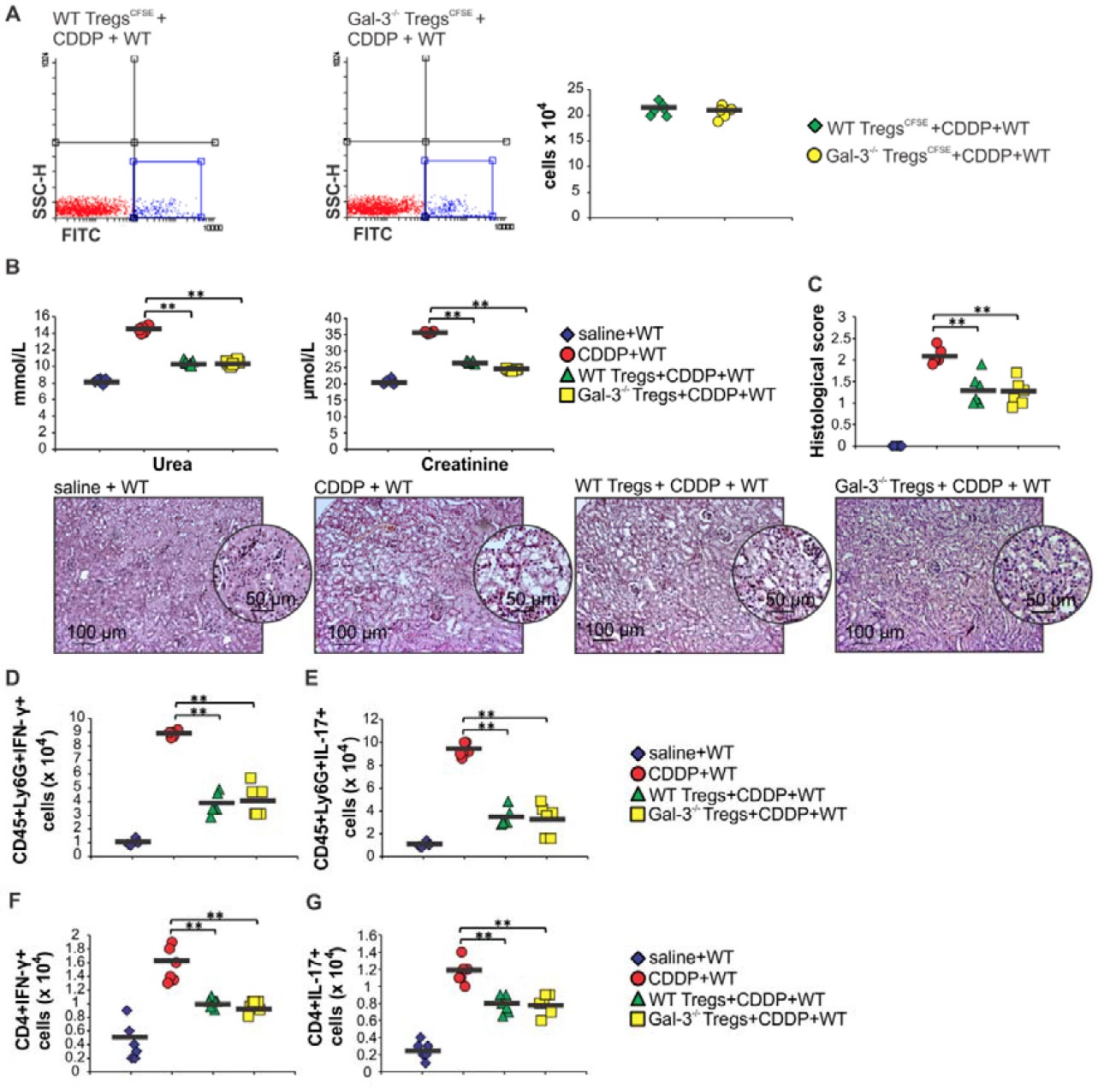
** Genetic deletion of Gal-3 did not alter migratory capacity and immunosuppressive properties of Tregs.** Tregs were fluorescence-labeled using pre-incubation with carboxyfluorescein diacetate succin-imidyl ester (CFSE). CSFE-labeled WT and Gal-3^-/-^Tregs were intravenously injected (1x10^6^ Tregs/mouse) in CDDP-treated WT mice and their number and function were evaluated by flow cytometry. Representative dotplots showing similar percentage and total number of CSFE-labeled WT and Gal-3^-/-^Tregs in the kidneys of CDDP-treated WT recipients. Engrafted WT and Gal-3^-/-^ Tregs managed to significantly attenuate CDDP-induced AKI, as evidenced by significantly lower serum levels of urea and creatinine (B) and reduced histological score (C), but there was no significant difference in the extent of AKI between CDDP-treated mice that received WT and Gal-3^-/-^Tregs, which was confirmed by representative kidney tissue sections. WT and Gal-3^-/-^ Tregs significantly reduced total number of IFN-γ and IL-17-producing neutrophils (5D-E) and CD4+ T cells (5F-G) but there was no significant difference in the total number of these cells between CDDP-treated mice that received WT and Gal-3^-/-^Tregs. Individual data points with Mean, obtained in one experiment with 6 mice per group; *p<0.05, **p<0.01,***p<0.001.

In order to determine whether genetic deletion of Gal-3 affected migratory capacity and immunosuppressive properties of Tregs, CSFE-labeled WT and Gal-3^-/-^Tregs were intravenously injected in CDDP-treated WT mice and their number and function were evaluated by flow cytometry (Figure 5 and Figure S3). As it is shown in Figure 5A similar number of CSFE-labeled WT and Gal-3^-/-^Tregs were detected in the kidneys of CDDP-treated WT recipients. Engrafted WT and Gal-3^-/-^ Tregs managed to significantly attenuate CDDP-induced AKI, as evidenced by significantly lower serum levels of urea and creatinine (p<0.01; Figure 5B) and reduced histological score (p<0.01; Figure 5C). Cellular-make-up of CDDP-injured kidneys revealed that WT and Gal-3^-/-^ Tregs significantly reduced total number of IFN-γ and IL-17-producing neutrophils (p<0.01; Figure 5D-E) and CD4+ T cells (p<0.01; Figure 5F-G). Importantly, there was no significant difference in nephroprotective effects and immunosuppressive capacity of WT and Gal-3^-/-^Tregs, suggesting that genetic deletion of Gal-3 did not directly affect migratory capacity and immunosuppressive properties of Tregs.

### Target disruption of Gal-3 significantly attenuated immunosuppressive capacity of renal-infiltrated DCs and enhanced their potential to generate detrimental Th1 and Th17 immune response in CDDP-injured kidneys

**Figure 6:**
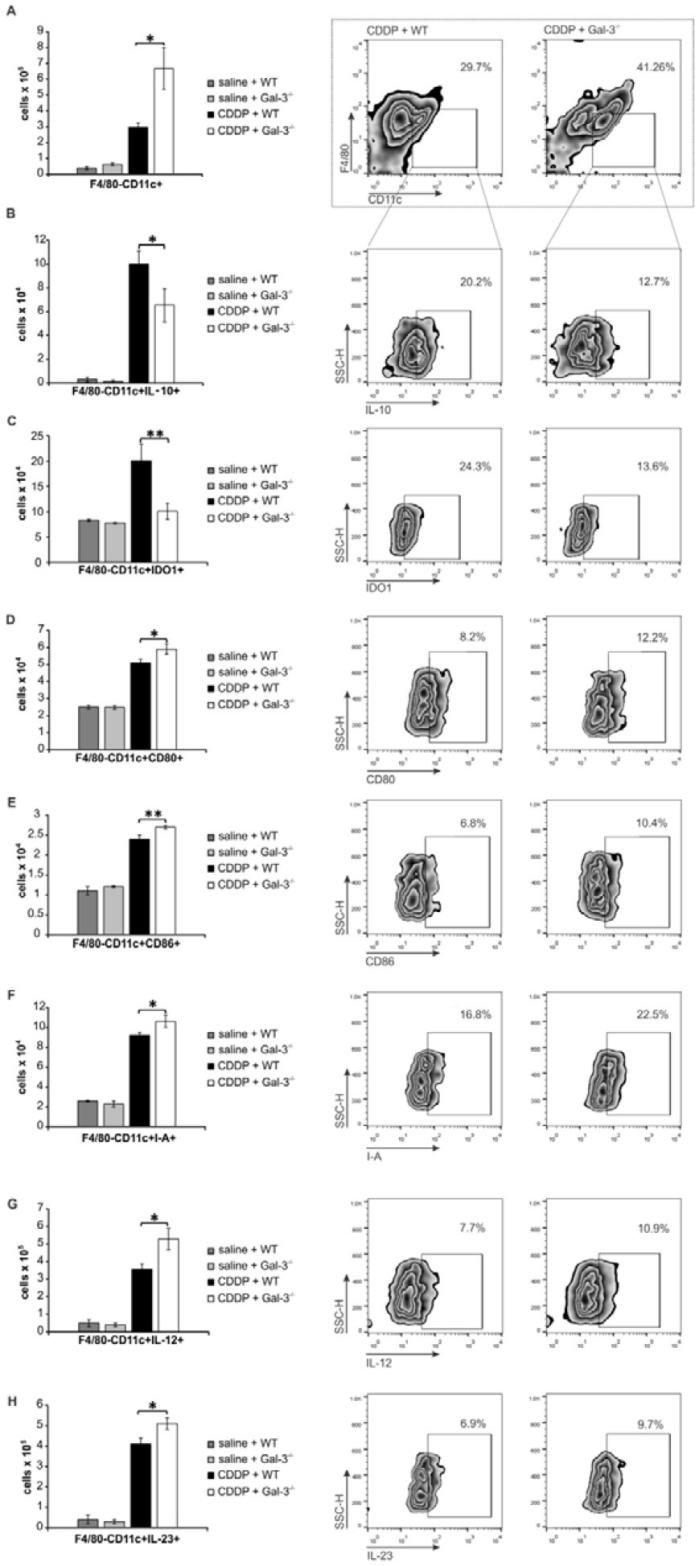
** Target disruption of Gal-3 significantly attenuated immunosuppressive capacity of renal-infiltrated DCs and enhanced their potential to generate detrimental Th1 and Th17 immune response in CDDP-injured kidneys.** Bar graphs (left panels) and representative density plots obtained by flow cytometry analysis and intracellular staining of renal-infiltrated DCs derived from saline or CDDP-treated WT and Gal-3^-/-^ mice, 72 h after CDDP administration (16 mg/kg body weight). Gal-3 deficiency resulted with increased presence of F4/80-CD11+DCs in the CDDP-injured kidneys (A). Representative density plots showing higher percentage of DCs in renal infiltrated cells of CDDP-treated Gal-3^-/-^ mice compared to similarly treated WT animals (A, right panel). DCs are determined as F4/80- CD11c+ renal infiltrated cells. Significantly lower number of IL-10-producing (B) and IDO1-expressing F4/80-CD11+DCs, but remarkably higher number of CD80-expressing (D), CD86-expressing (E), I-A-expressing (F), IL-12-producing (G) and IL-23-producing F4/80-CD11+DCs were noticed in the kidneys of CDDP-treated Gal-3^-/-^ mice. Representative density plots showing percentages of IL-10-producing, IDO1-, CD80-, CD86-, I-A-expressing, IL-12 and IL-23-producing cells, gated in the population of F4/80-CD11c+ renal DCs isolated from the kidneys of CDDP-treated WT and Gal-3^-/-^ mice, 72 h after CDDP injection (B-G, right panels). Data from two individual experiments with 8 mice per group are shown as Mean ± SEM; *p<0.05, **p<0.01.

In order to determine whether Gal-3 deficiency altered function of renal-infiltrated DCs which guide T cell-driven immune response in CDDP-induced nephrotoxicity 6, we analyzed phenotype of renal-infiltrated F4/80-CD11c+DCs in WT and Gal-3^-/-^ mice. F4/80-CD11c+DCs were present in significantly higher number in kidneys of CDDP-treated Gal-3^-/-^ mice (Figure 6A, p<0.05). Intracellular staining revealed that target disruption of Gal-3 significantly attenuated capacity of renal-infiltrated DCs to produce renoprotective and immunosuppressive IL-10 (Figure 6B, p<0.05) and IDO1 (Figure 6C, p<0.01) and enhanced their capacity to generate detrimental Th1 and Th17 immune response in CDDP-injured kidneys (Figure 6D-H). Significantly higher number of DCs that expressed co-stimulatory CD80 (Figure 6D, p<0.05) and CD86 molecules (Figure 6E, p<0.01), major histocompatibility class II (I-A) molecule (Figure 6F, p<0.05), IL-12-producing (Figure 6G, p<0.05) as well as IL-23-producing DCs (Figure 6H, p<0.05) were noticed in the kidneys of CDDP-treated Gal-3^-/-^ mice compared to WT animals.

### Gal-3 is required for TLR-2-dependent activation of IDO1/KYN pathway in renal DCs and consequent generation of immunosuppressive Tregs

**Figure 7:**
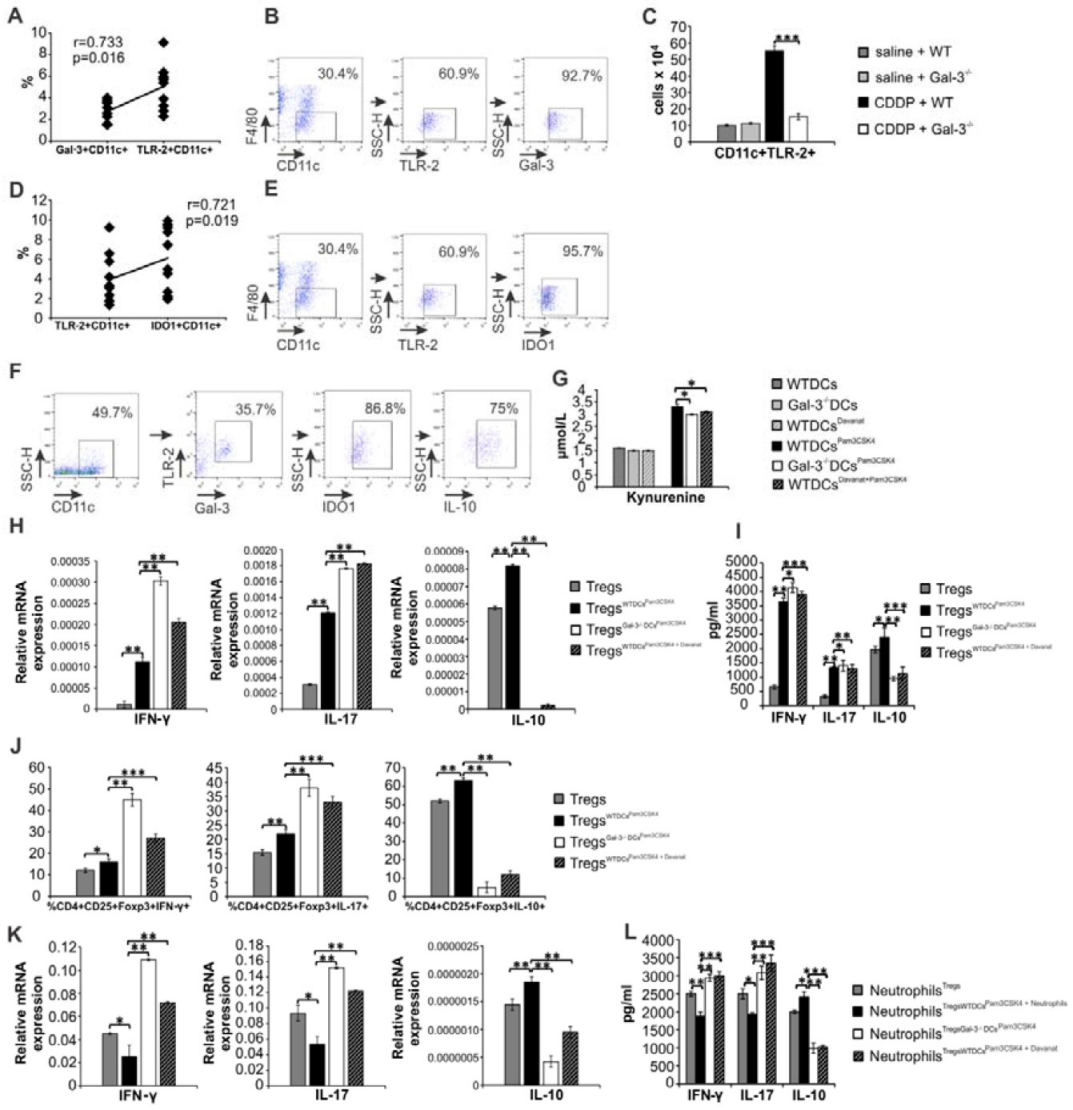
** Gal-3 is required for TLR-2-dependent activation of IDO1/KYN pathway in renal DCs and consequent generation of immunosuppressive Tregs.** There was strong positive correlation between percentages of Gal-3+CD11c+DCs and TLR-2+CD11c+DCs in the kidneys (A). Representative flow cytometry dot plots showing expression of Gal-3 in TLR-2+ cells previously gated in the population of F4/80-CD11c+ renal DCs (B). Significantly lower number of TLR-2-expressing CD11c+DCs was observed in the kidneys of CDDP-treated Gal-3^-/-^ mice compared to CDDP-treated WT animals, 72 h after CDDP injection (16 mg/kg body weight). There was strong positive correlation between percentages of IDO1+CD11c+DCs and TLR-2+CD11c+DCs in the kidneys (D). Representative flow cytometry dot plots showing expression of IDO1 in TLR-2+ cells previously gated in the population of F4/80-CD11c+ renal DCs (E). Representative dot plots showing that majority of TLR-2+Gal-3+IDO1+DCs express IL-10 (F). Significantly lower concentrations of KYN were noticed in supernatants of Gal-3^-/-^DCs^Pam3CSK4^ and WTDCs^PAM3CSK4+Davanat^ compared to WTDCs^Pam3CSK4^ (G). Real-time PCR gene analysis (H), ELISA (I) and flow cytometry (J) results showing expression and production of inflammatory IFN-γ, IL-17, and anti-inflammatory IL-10 in Tregs (H, J) and appropriate supernatants (I) before and after culturing with WTDCs^Pam3CSK4^, Gal-3-/-DCs^Pam3CSK4^ or WT DCs^PAM3CSK4+Davanat^ in contact-independent manner within transwell system (ratio between DCs and Tregs was 1:10). Real-time PCR gene analysis (K) and ELISA (L) results showing expression and production of inflammatory IFN-γ, IL-17 and anti-inflammatory IL-10 in LPS-activated neutrophils that were cultured with Tregs previously stimulated by WTDCs^Pam3CSK4^, Gal-3^-/-^DCs^Pam3CSK4^ or WT DCs^PAM3CSK4+Davanat^. Transwell systems were used to separate neutrophils and Tregs and ratio between neutrophils and Tregs was 10:1. Data from two individual experiments with 8 mice per group are shown as Mean ± SEM; *p<0.05, **p<0.01;***p<0.001.

It is well known that CDDP treatment induces increased expression of renoprotective TLR-2 in injured kidneys 3 and that DCs suppress Th1 and Th17 driven inflammation by promoting expansion of Tregs in IDO1/KYN-dependent manner 57. Accordingly, we evaluated the importance of Gal-3 for TLR2-dependent immunosuppressive function of renal DCs in CDDP-induced AKI. Firstly, we noticed strong positive correlation between percentages of Gal-3+CD11c+DCs and TLR-2+CD11c+DCs in the kidneys (Figure 7A; r=0.733). Gal-3 was expressed on the membrane of the majority of TLR-2+F4/80-CD11c+renal DCs (Figure 7B). CDDP-provoked expression of TLR-2 on renal DCs was remarkably reduced in Gal-3^-/-^ mice (p<0.001; Figure 7C), indicating importance of Gal-3 for TLR-2-dependent signaling in renal DCs. In line with these findings, there was strong positive correlation between percentages of IDO1+CD11c+DCs and TLR-2+CD11c+DCs in the kidneys (Figure 7D; r=0.721). IDO1 was expressed in the majority of TLR-2+F4/80-CD11c+renal DCs (Figure 7E). Intracellular staining revealed that majority of TLR-2 and Gal-3-expressing renal DCs express IDO1 and most of TLR-2+Gal-3+IDO1+DCs express immunosuppressive IL-10 (Figure 7F).

In order to confirm importance of Gal-3 for TLR-2-dependent activation of immunosuppressive IDO1/KYN pathway in DCs, we analyzed the difference in KYN production between TLR-2-primed WTDCs (WTDCs^Pam3CSK4^) and WTDCs^Pam3CSK4^ that were cultured in the presence of selective Gal-3 inhibitor-Davanat (WTDCs^Pam3CSK4+Davanat^) (Figure 7G). Activation of TLR-2 significantly enhanced production of immunosuppressive KYN in renal DCs (Figure 7G). Importantly, genetic deletion as well as pharmacological inhibition of Gal-3, remarkably reduced TLR-2-dependent secretion of KYN in WTDCs (Figure 7G). Significantly lower concentrations of KYN were measured in supernatants of Gal-3^-/-^DCs^Pam3CSK4^ and WTDCs^PAM3CSK4+Davanat^ compared to WTDCs^Pam3CSK4^ (Figure 7G, p<0.05).

Genetic deletion as well as pharmacological inhibition of Gal-3 in WTDCs^Pam3CSK4^ diminished their capacity to maintain immunosuppressive phenotype of Tregs and to prevent trans-differentiation of Tregs in Th1 or Th17 cells (Figure 7H-J). Decreased expression and production of anti-inflammatory IL-10 (p<0.01) and increased expression and production of inflammatory IFN-γ (p<0.01) and IL-17 (p<0.01) were noticed in CD4+CD25+FoxP3+Tregs which had been cultured with Gal-3^-/-^DCs^Pam3CSK4^ or WT DCs^PAM3CSK4+Davanat^ compared to Tregs that were cultured alone or with WTDCs^Pam3CSK4^ (Figure 7H-J; Figure S4).

Additionally, Tregs generated by Gal-3^-/-^DCs^Pam3CSK4^ or WTDCs^Pam3CSK4+Davanat^ were not able to optimally suppress expression and production of IFN-γ and IL-17 (p<0.05 for IFN-γ, p<0.01 for IL-17; Figure 7K-L) or to induce enhanced expression and production of IL-10 (p<0.01, Figure 7K-L) in activated neutrophils, indicating the importance of Gal-3 for DC-driven regulation of cross-talk between Tregs and neutrophils.

### Intravenous injection of TLR-2 primed WTDCs significantly attenuated CDDP-induced AKI by promoting expansion of immunosuppressive Tregs in IDO1/KYN-dependent manner

**Figure 8:**
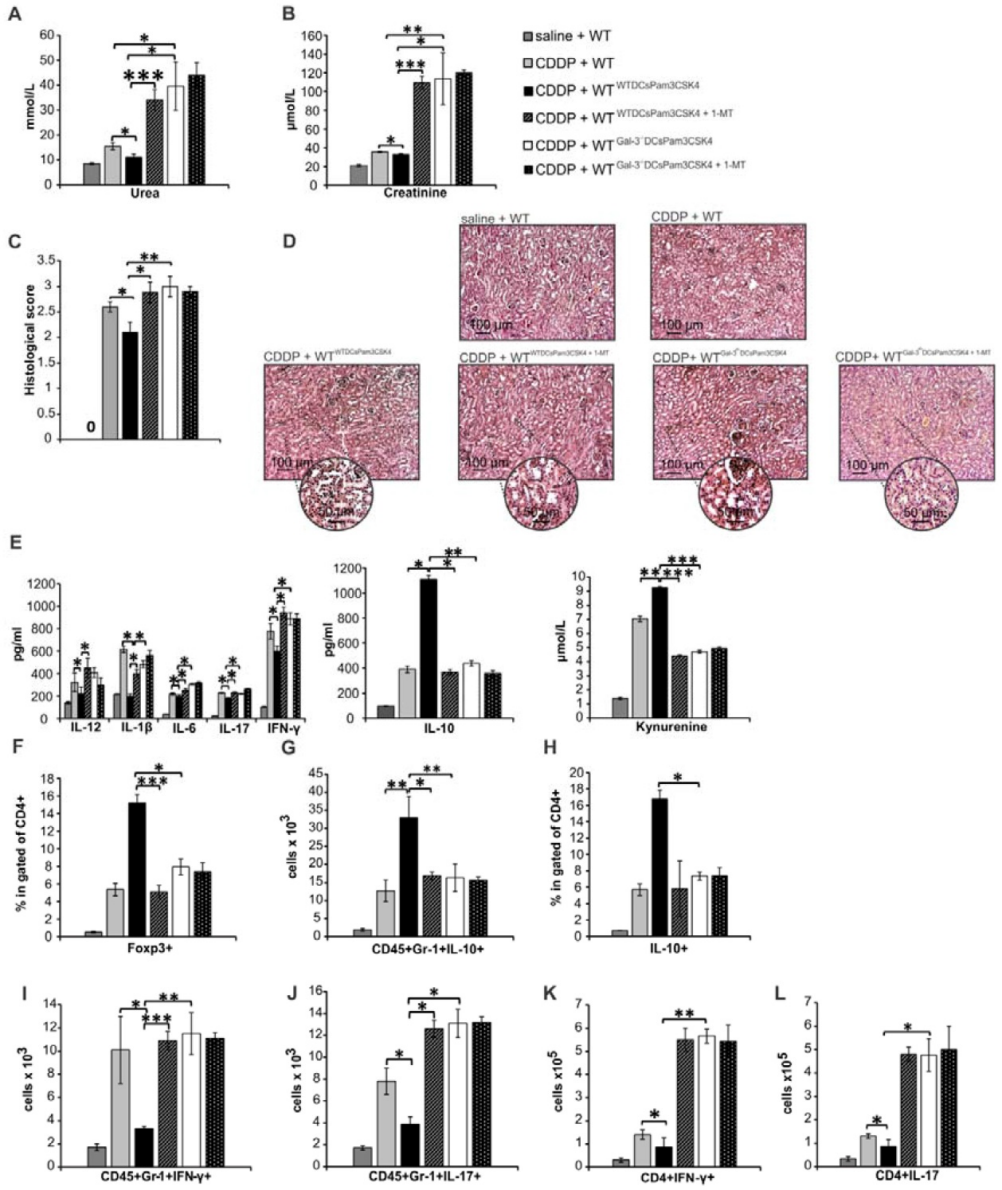
** Transfer of WTDCs^Pam3CSK4^ significantly attenuated CDDP-induced AKI in WT recipients by promoting expansion of immunosuppressive Tregs in IDO1/KYN-dependent manner.** TLR-2-primed DCs, isolated from the kidneys of untreated WT and Gal‐3^-/-^ mice (WTDCs^Pam3CSK4^ and Gal‐3^-/-^DCs^Pam3CSK4^), were intravenously injected (5×10^5^cells/mouse) in CDDP-treated WT recipients (WT^WTDCsPam3CSK4^ and WT^Gal‐3-/-DCsPam3CSK4^) two days prior CDDP administration (16 mg/kg body weight). IDO1 was inhibited in TLR-2-primed renal DCs (WTDCs^Pam3CSK4+1-MT^) and Gal-3^-/-^DCs (Gal-3^-/-^DCs^Pam3CSK4+1-MT^) by using 1-methyl tryptophan (1-MT; 2 mM). Transfer of WTDCs^Pam3CSK4^ managed to attenuate CDDP-injured AKI and inflammation in CDDP-treated WT recipients, as evidenced by notably lower serum levels of urea (A), creatinine (B), decreased histological score (C), reduced extent of renal injury in the cortex of CDDP-injured kidneys (D), decreased serum levels of inflammatory, Th1/Th17-related cytokines and increased serum levels of immunosuppressive IL-10 and KYN (E), significantly higher presence of Tregs (F), and IL-10-producing neutrophils (G) and CD4+T cells (H), but notably decreased of IFN-γ (I) and IL-17-producing neutrophils (J) as well as IFN-γ (K) and IL-17-producing CD4+T cells (L). Transfer of WTDCs^Pam3CSK4+1-MT^ or Gal-3^-/-^DCs^Pam3CSK4^ did not expand immunosuppressive Tregs (F), IL-10-producing neutrophils (G) and CD4+Tcells (H) in CDDP-injured kidneys, but promoted polarization of renal-infiltrating neutrophils (I-J) and CD4+T cells (K-L) towards IFN-γ-producing (I, K) and IL-17-producing cells (J, L), resulting in significant aggravation of CDDP-induced AKI in WT recipients (A-D). There was no significant difference between CDDP-treated WT recipients of Gal-3^-/-^DCs^Pam3CSK4+1-MT^ and Gal-3^-/-^DCs^Pam3CSK4^ (A-L). Data from two individual experiments with 8 mice per group are shown as Mean ± SEM; *p<0.05, **p<0.01;***p<0.001.

In order to demonstrate crucial role of Gal-3 for DCs^Pam3CSK4^-dependent modulation of CDDP-induced AKI, we injected WTDCs^Pam3CSK4^ or Gal-3^-/-^DCs^Pam3CSK4^ in CDDP-treated WT or Gal-3^-/-^ recipients. Transfer of WTDCs^Pam3CSK4^ attenuated CDDP-injured AKI and inflammation in CDDP-treated WT recipients (WT^WTDCsPam3CSK4^), as evidenced by notably lower serum levels of urea (Figure 8A, p<0.05), creatinine (Figure 8B, p<0.05) and inflammatory cytokines (IL-12, IL-1β, IL-6, IL-17, IFN-γ, p<0.05, Fig.8E left panel). Significantly reduced histological score (Figure 8C) and preserved renal architecture (Figure 8D), seen in CDDP-treated WT^WTDCsPam3CSK4^ mice, were accompanied by elevated serum levels of immunosuppressive IL-10 (Figure 8E middle panel, p<0.05) and KYN (Figure 8E right panel, p<0.01), remarkable expansion of renal-infiltrated CD4+FoxP3+Tregs (Figure 8F, p<0.001), IL-10-producing neutrophils (Figure 8G, p<0.01) and CD4+ T cells (Figure 8H, p<0.05) and significantly lower number of IFN-γ and IL-17-producing neutrophils (Figure 8I-J, p<0.05) and CD4+ T lymphocytes (Figure 8K-L, p<0.05).

On the contrary, single intravenous injection of Gal-3^-/-^DCs^Pam3CSK4^ notably aggravated CDDP-induced AKI in WT recipients (WT^Gal-3-/-DCsPam3CSK4^), as evidenced by significantly increased serum levels of urea (Figure 8A, p<0.01), creatinine (Figure 8B, p<0.05), histological score (Figure 8C, p<0.05) and massive destruction of proximal tubules (Figure 8D). As it is shown in Figure 8E, consistent with the deterioration of kidney function, significantly increased serum levels of inflammatory, pro-Th1 (IL-12, p<0.05) and pro-Th17 cytokines (IL-1β, p<.0.05; IL-6, p<0.05) as well as IFN-γ (p<0.05) and IL-17 (p<0.05) were found in WT^Gal-3-/-DCsPam3CSK4^ mice (Figure 8E, left panel). Consistently, cellular make-up of the kidneys revealed significantly increased total number of IFN-γ and IL-17 producing neutrophils (Figure 8I-J, p<0.05) and CD4+ T cells (Figure 8K-L, p<0.01 for Th1 and p<0.05 for Th17 cells) in the CDDP-injured kidneys of WT^Gal-3-/-DCsPam3CSK4^ mice, indicating that transfer of Gal-3^-/-^DCs^Pam3CSK4^ aggravated AKI in CDDP-treated WT recipients by enhancing IFN-γ and IL-17-driven inflammation.

**Figure 9:**
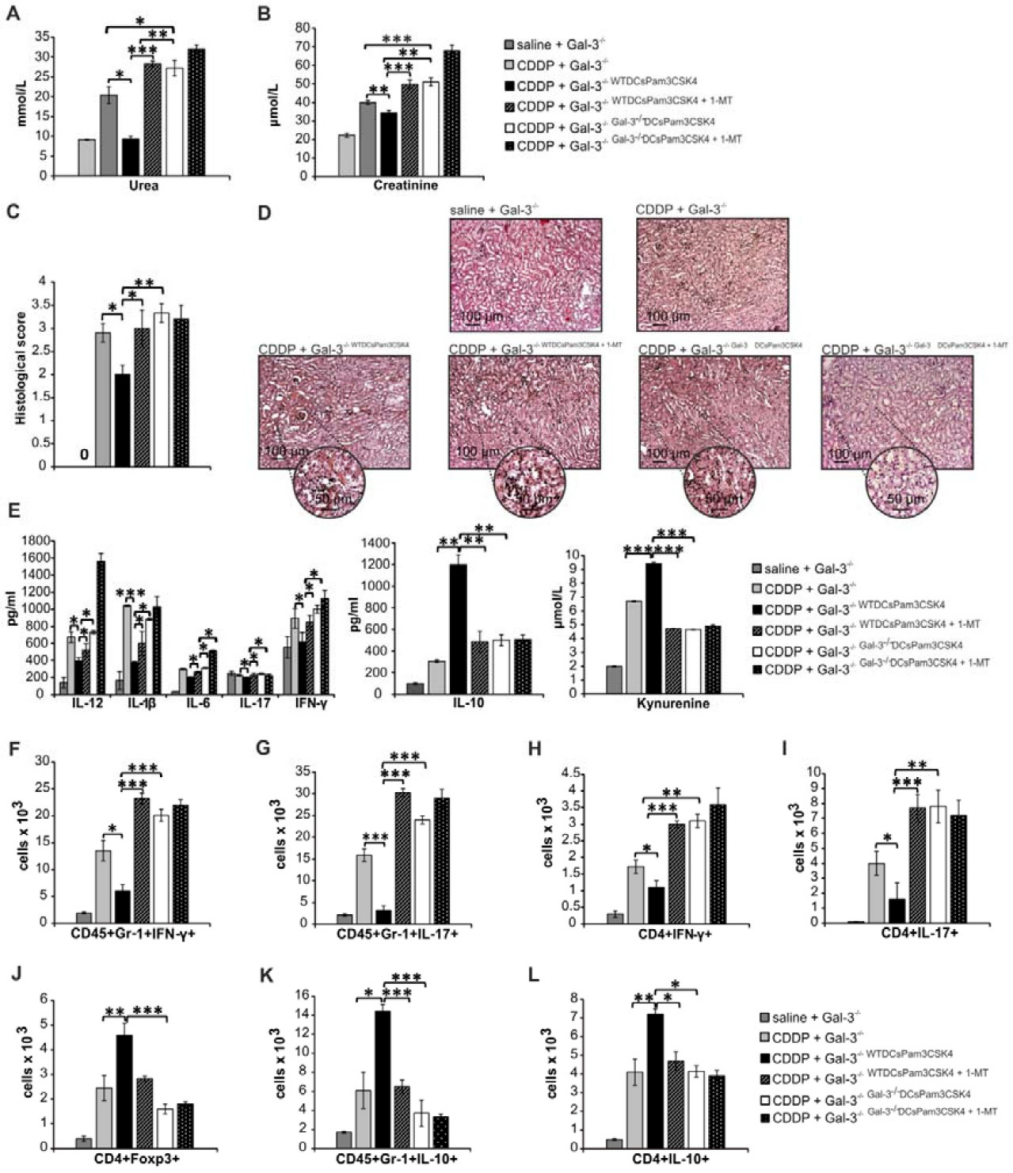
** Transfer of WTDCs^Pam3CSK4^ significantly attenuated CDDP-induced AKI in Gal-3^-/-^ recipients by promoting expansion of immunosuppressive Tregs in IDO1/KYN-dependent manner.** TLR-2-primed DCs, isolated from the kidneys of untreated WT and Gal‐3^-/-^ mice (WTDCs^Pam3CSK4^ and Gal‐3^-/-^DCs^Pam3CSK4^), were intravenously injected (5×10^5^cells/mouse) in CDDP-treated Gal-3^-/-^ recipients (Gal-3^-/-WTDCsPam3CSK4^ and Gal-3^-/-Gal‐3-/-DCsPam3CSK4^) two days prior CDDP administration (16 mg/kg body weight). IDO1 was inhibited in TLR-2-primed renal DCs (WTDCs^Pam3CSK4+1-MT^) and Gal-3^-/-^DCs (Gal-3^-/-^DCs^Pam3CSK4+1-MT^) by using 1-methyl tryptophan (1-MT; 2 mM). Intravenous injection of WTDCs^Pam3CSK4^ attenuated CDDP-injured AKI and inflammation in CDDP-treated Gal-3^-/-^ recipients, as evidenced by notably lower serum levels of urea (A), creatinine (B), decreased histological score (C), reduced extent of renal injury in the cortex of CDDP-injured kidneys (D), decreased serum levels of inflammatory, Th1/Th17-related cytokines and increased serum levels of immunosuppressive IL-10 and KYN (E), significantly reduced total number of IFN-γ (F) and IL-17-producing neutrophils (G), IFN-γ-(H) and IL-17-producing CD4+T cells (J), but remarkably increased number of immunosuppressive Tregs (J), IL-10-producing neutrophils (K) and CD4+T cells (L). Transfer of WTDCs^Pam3CSK4+1-MT^ or Gal-3^-/-^DCs^Pam3CSK4^ did not expand immunosuppressive Tregs (J), IL-10-producing neutrophils (K) and CD4+Tcells (L) in CDDP-injured kidneys, but promoted polarization of renal-infiltrating neutrophils (F-G) and CD4+T cells (H-I) towards IFN-γ-producing (F, H) and IL-17-producing cells (G, I), resulting in significant aggravation of CDDP-induced AKI in Gal-3^-/-^ recipients (A-D). There was no significant difference between CDDP-treated WT recipients of Gal-3^-/-^DCs^Pam3CSK4+1-MT^ and Gal-3^-/-^DCs^Pam3CSK4^ (A-L). Data from two individual experiments with 8 mice per group are shown as Mean ± SEM; *p<0.05, **p<0.01;***p<0.001.

In similar manner as it was observed in CDDP-injured WT recipients (Figure 8; Figure S5), injection of Gal-3^-/-^DCs^Pam3CSK4^ significantly aggravated renal failure in CDDP-treated Gal-3^-/-^ recipients (Gal-3^-/-Gal-3-/-DCsPam3CSK4^) by promoting IFN-γ and IL-17-driven inflammation (Figure 9A-L; Figure S6). Importantly, transfer of WTDCs^Pam3CSK4^ completely attenuated AKI in CDDP-injured Gal-3^-/-^ recipients (Gal-3^-/-WTDCsPam3CSK4^), confirming that expression of Gal-3 on DCs was crucially responsible for DC^Pam3CSK4^-mediated amelioration of CDDP-induced nephrotoxicity (Figure 9). Significantly lower serum concentrations of urea and creatinine (p<0.05 for urea and p<0.01 for creatinine; Figure 9A-B) and notably reduced histological score were noticed in CDDP-treated Gal-3^-/-WTDCsPam3CSK4^ mice (Figure 9C). CDDP-caused massive necrosis of proximal tubules, cast formation and erythrocyte congestion, observed in Gal-3^-/-^ animals, were not seen in the kidneys of CDDP-treated Gal-3^-/-WTDCsPam3CSK4s^ mice (Figure 9D). Biochemical and histological findings were accompanied by significantly lower serum levels of Th1 and Th17-related cytokines (p<0.05; Figure 9E, left panel) and remarkably reduced presence of IFN-γ and IL-17 producing neutrophils (p<0.05; Figure 9F-G) and CD4+ T cells (p<0.05; Figure 9H-I) in the kidneys of CDDP-treated Gal-3^-/-WTDCsPam3CSK4^ mice. Transfer of WTDCs^Pam3CSK4^ significantly elevated serum levels of nephroprotective IL-10 and KYN (p<0.01 for IL-10 and p<0.001 for KYN; Figure 7E middle and right panels) that was followed by remarkable increase in total number of immunosuppressive CD4+FoxP3+Tregs (p<0.01; Figure 9J), IL-10-producing neutrophils (p<0.05; Figure 9K) and CD4+ T cells (p<0.01 Figure 9L) in the kidneys of CDDP-treated Gal-3^-/-WTDCsPam3CSK4^ mice.

Importantly, 1-MT-induced inhibition of IDO1 activity in TLR-2-primed WTDCs (WTDCs^Pam3CSK4+1-MT^) completely abrogated their capacity to attenuate CDDP-induced AKI in CDDP-treated WT (WT^WTDCsPam3CSK4+1-MT^; Figure 8) and Gal-3^-/-^(Gal-3^-/-WTDCsPam3CSK4+1-MT^; Figure 9) recipients. Transfer of WTDCs^Pam3CSK4+1-MT^ failed to promote expansion of immunosuppressive CD4+FoxP3+Tregs (Figure 8J and Figure 9J) and to prevent their trans-differentiation in Th1 and Th17 cells (Figure 8K-L and Figure 9H-I). Additionally, reduced presence of Tregs was accompanied with significantly lower number of IL-10-producing neutrophils (Figure 8H and Figure 9K) and increased number of IFN-γ (p<0.001; Figure 8I and Figure 9F) and IL-17 producing neutrophils (p<0.05; Figure 8J and p<0.001; Figure 9G), indicating that activation of IDO1/KYN pathway was crucially important for Gal-3-dependent immunosuppressive effects of renal DCs^Pam3CSK4^ and for DCs^Pam3CSK4^-induced expansion of Tregs.

In order to determine whether Gal-3 and IDO1 are members of the same TLR-2-initiated signaling pathway responsible for DCs-mediated attenuation of CDDP-induced AKI, we inhibited IDO1 activity in Gal-3 deficient TLR-2-primed DCs (Gal-3^-/-^DCs^Pam3CSK4+1-MT^) and analyzed their nephroprotective and immunosuppressive effects in CDDP-treated WT (Figure 8) and Gal-3^-/-^ recipients (Figure 9). There was no significant difference in serum levels of urea and creatinine (Figure 8A and Figure 9A), extent of renal injury (Figure 8C-D and Figure 9C-D), serum concentration of inflammatory and immunosuppressive cytokines (Figure 8E and Figure 9E), total number of renal-infiltrated IFN-γ and IL-17-producing neutrophils (Figure 8I-J and Figure 9F-G), Th1 and Th17 lymphocytes (Figure 8K-L and Figure 9H-I) and IL-10-producing neutrophils and Tregs (Figure 8F-H and Figure 9J-L) between CDDP-treated WT and Gal-3^-/-^ recipients of Gal-3^-/-^DCs^Pam3CSK4+1-MT^ and Gal-3^-/-^DCs^Pam3CSK4^. Thus, inhibition of IDO1 activity did not significantly alter immunomodulatory properties of TLR-2-primed Gal-3^-/-^DCs, suggesting that Gal-3 and IDO1 are members of the same TLR-2-initiated signaling pathway which was responsible for nephroprotective and immunosuppressive effects of TLR-2-primed renal DCs in attenuation of CDDP-induced AKI.

### Expression of Gal-3 on TLR-2-primed DCs is crucially important for their capacity to enhance nephroprotective and immunosuppressive properties of Tregs

**Figure 10:**
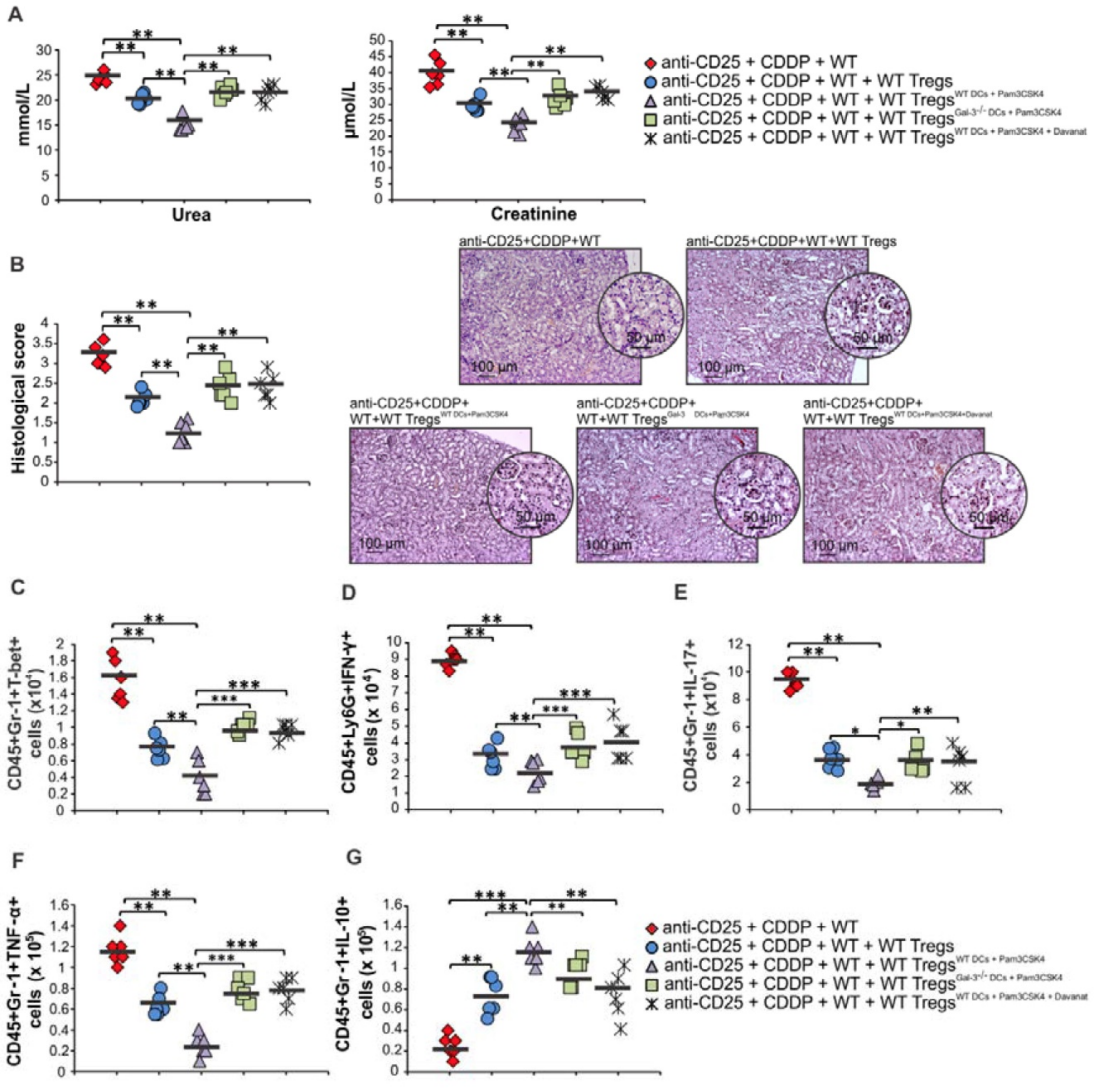
** Expression of Gal-3 on TLR-2-primed DCs is crucially important for their capacity to enhance nephroprotective and immunosuppressive properties of Tregs.** For the depletion of Tregs, anti-CD25 monoclonal antibody (250 μg/mouse) was intraperitoneally given to mice 3 days before CDDP administration (16 mg/kg body weight). For transfer experiments non-primed Tregs, Tregs primed with WTDC^Pam3CSK4^, WTDC^Pam3CSK4+Davanat^ or Gal-3^-/-^ DC^Pam3CSK4^ (1x10^6^ Tregs/ mouse) were intravenously injected in CDDP-treated animals 18 h before induction of AKI. Significantly lower serum levels of urea and creatinine (A) and reduced histological score accompanied with attenuated renal injury, observed in the cortex of CDDP-injured kidneys (B) were observed in Treg-depleted CDDP-treated mice that received WTDC^Pam3CSK4^-primed Tregs compared to the Treg-depleted CDDP-injured animals that received non-primed Tregs. There was significantly lower number of T-bet-expressing, IFN-γ-, IL-17- and TNF-α-producing neutrophils (C-F) and remarkably higher number of IL-10-producing neutrophils (G) in the kidneys of Treg-depleted CDDP-treated mice that received WTDC^Pam3CSK4^-primed Tregs. Significantly higher serum concentrations of urea and creatinine (A), more severe injury of proximal tubular epithelial cells and increased histological score (B), significantly higher number of inflammatory neutrophils (C-F) and significantly reduced number of immunosuppressive, IL-10-producing neutrophils (G) in the kidneys of Treg-depleted CDDP-treated mice that received Gal-3^-/-^DC^Pam3CSK4^ or WTDC^Pam3CSK4+Davanat^ -primed Tregs compared to Treg-depleted CDDP-treated mice that received WTDC^Pam3CSK4^-primed Tregs. Individual data points with Mean, obtained in one experiment with 6 mice per group; *p<0.05, **p<0.01,***p<0.001.

It is well known that Tregs suppress CDDP-induced acute renal inflammation and that depletion of Tregs results in exacerbation of AKI 9. In order to confirm crucial importance of Gal-3:TLR-2 axis for DC-dependent enhancement of nephroprotective and immunosuppressive properties of Tregs, we intravenously injected non-primed Tregs, Tregs primed with WTDC^Pam3CSK4^, WTDC^Pam3CSK4+Davanat^ or Gal-3^-/-^ DC^Pam3CSK4^ in Treg-depleted CDDP-treated WT mice (Figure 10; Figure S7). WTDC^Pam3CSK4^ significantly enhanced capacity of Tregs for attenuation of CDDP-induced AKI and inflammation. Significantly lower serum levels of urea and creatinine (p<0.01; Figure 10A) and reduced histological score (p<0.01; Figure 10B) were observed in Treg-depleted CDDP-treated mice that received WTDC^Pam3CSK4^-primed Tregs compared to the Treg-depleted CDDP-injured animals that received non-primed Tregs. Histological analysis revealed preserved architecture of renal tissue and paucity of renal-infiltrated inflammatory cells in the kidneys of Treg-depleted CDDP-treated mice that received WTDC^Pam3CSK4^-primed Tregs (Figure 10B). Additionally, WTDC^Pam3CSK4^ significantly enhanced capacity of Tregs to inhibit production of inflammatory cytokines (IFN-γ, IL-17, TNF-α) and to promote production of immunosuppressive IL-10 in renal-infiltrated neutrophils, as evidenced by significantly lower number of T-bet-expressing, IFN-γ-, IL-17- and TNF-α-producing neutrophils (p<0.01; Figure 10C-F) and remarkably higher number of IL-10-producing neutrophils (p<0.001; Figure 10G) in the kidneys of Treg-depleted CDDP-treated mice that received WTDC^Pam3CSK4^-primed Tregs. Importantly, genetic deletion or pharmacological inhibition of Gal-3 remarkably attenuated capacity of TLR-2-primed DCs to enhance nephroprotective and immunosuppressive properties of Tregs, as evidenced by significantly higher serum concentrations of urea and creatinine (p<0.01; Figure 10A), more severe injury of proximal tubular epithelial cells and increased histological score (p<0.01; Figure 10B), significantly higher number of inflammatory neutrophils (p<0.05 for IL-17-producing and p<0.001 for T-bet-expressing, IFN-γ- and TNF-α-producing CD45+Gr-1+cells; Figure 10C-F) and significantly reduced number of immunosuppressive, IL-10-producing neutrophils (p<0.01; Figure 10G) in the kidneys of Treg-depleted CDDP-treated mice that received Gal-3^-/-^DC^Pam3CSK4^ or WTDC^Pam3CSK4+Davanat^ -primed Tregs compared to Treg-depleted CDDP-treated mice that received WTDC^Pam3CSK4^-primed Tregs.

### Depletion of Tregs diminished Gal-3-dependent capacity of TLR-2-primed renal DCs to suppress IFN-γ and IL-17 driven inflammation in CDDP-injured kidneys

**Figure 11:**
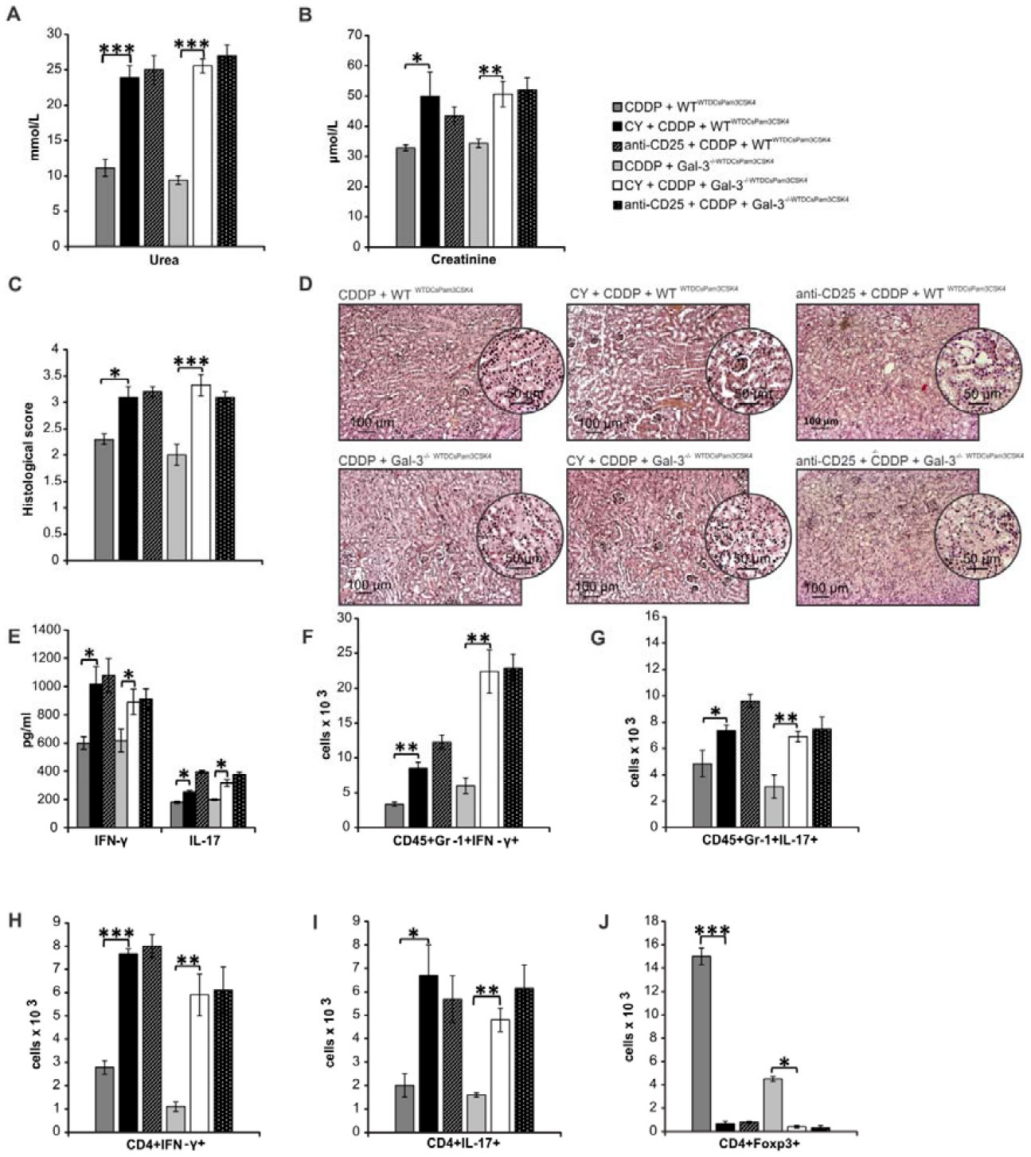
** Depletion of Tregs completely diminished Gal-3-dependent capacity of TLR-primed renal DCs to suppress IFN-γ and IL-17 driven inflammation in CDDP-injured kidneys.** For the depletion of Tregs, CDDP-treated WT^WTDCPam3CSK4^ and Gal-3^-/-WTDCPam3CSK4^ mice received either cyclophosphamide (CY; 10 mg/kg) 3 days before CDDP administration (16 mg/kg body weight) or anti-CD25 (P61) monoclonal antibody (250 μg/mouse). Significantly elevated serum levels of urea (A) and creatinine (B), increased histological score (C), more severe tubular epithelial cell injury (D), elevated serum levels of IFN-γ and IL-17 (E), increased presence of IFN-γ- and IL-17-producing neutrophils (F-G), and IFN-γ- and IL-17-producing CD4+T cells (H-I) were noticed in the kidneys of CY+CDDP- and anti-CD25+CDDP-treated WT and Gal-3^-/-^ recipients of WTDCs^Pam3CSK4^ compared to CDDP-treated WT^WTDCsPam3CSK4^ and Gal-3^-/-WTDCsPam3CSK4^ that were not depleted from Tregs (J). Data from two individual experiments with 8 mice per group are shown as Mean ± SEM; *p<0.05, **p<0.01;***p<0.001.

In order to determine the importance of Tregs for Gal-3-dependent nephroprotective and immunosuppressive function of TLR-2-primed renal DCs, we analyzed the effects of CY or anti-CD25 antibody-induced depletion of Tregs in CDDP-treated WT^WTDCsPam3CSK4^ and Gal-3^-/-WTDCsPam3CSK4^mice (Figure 11; Figure S8). Depletion of Tregs completely abrogated Gal-3-dependent nephroprotective and immunosuppressive effects of WTDCs^Pam3CSK4^ (Figure 11A-D). Significantly increased serum levels of urea, creatinine (p<0.05; Figure 11A-B), histological score (Figure 11C) and massive tubular injury (Figure 11D) were observed in CY+CDDP- and anti-CD25+CDDP-treated WT^WTDCsPam3CSK4s^ and Gal-3^-/-WTDCsPam3CSK4s^mice. Significantly elevated serum concentrations of IFN-γ and IL-17 (p<0.05; Figure 11E) and remarkably higher presence of IFN-γ and IL-17 producing neutrophils (Figure 11F-G, p<0.05) and CD4+ T cells (Figure 11H-I, p<0.01 for Th1 and p<0.05 for Th17 cells) were observed in the kidneys of CY+CDDP- and anti-CD25+CDDP-treated WT^WTDCsPam3CSK4^ and Gal-3^-/-WTDCsPam3CSK4^mice, indicating that depletion of Tregs diminished Gal-3-dependent capacity of TLR-2-primed DCs to suppress IFN-γ and IL-17 driven inflammation in AKI.

## Discussion

Renal DCs represent specific sub-population of resident kidney immune cells which acquire distinct phenotypic and functional characteristics depending on the intrarenal inflammatory conditions, having pro-inflammatory and pathogenic role in T cell-mediated glomerulonephritis and lupus nephritis, while playing immunosuppressive and nephroprotective role in CDDP-induced AKI 4. Under the influence of DAMPs and alarmins, which are released from CDDP-injured PTECs, renal DCs acquire tolerogenic phenotype and immunosuppressive characteristics which enable them to elicit strong anti-inflammatory and reparative response in the CDDP-injured kidneys 4. Accordingly, depletion of renal DCs significantly aggravated tubular injury and renal dysfunction and considerably reduced survival of CDDP-treated mice 5.

The role of Gal-3 for migration and antigen-presenting function of bone marrow-derived DCs has already been described by us and others 12-13, 18-19, 42, 58-59. Interestingly, renal DCs and bone marrow-derived DCs significantly differ in their capacity to produce immunosuppressive cytokines which are responsible for DC-dependent attenuation of CDDP-caused renal injury and inflammation 5-6, 60. In line with these findings, we provide here the first evidence on the importance of Gal-3 for immunosuppressive effects of renal DCs in alleviation of CDDP-caused renal injury and inflammation. Genetic deletion as well as pharmacological inhibition of Gal-3 significantly impaired capacity of renal DCs to induce TLR-2:IDO-1-dependent expansion of renal-infiltrating Tregs and to initiate reparative response in CDDP-injured kidneys (Figure 4-7). Accordingly, remarkably reduced total number of immunosuppressive, IL-10 producing neutrophils (Figure 2E), M2 macrophages (Figure 2I) and Tregs (Figure 3G), but increased presence of inflammatory M1 macrophages (Figure 2H), IFN-γ or IL-17-producing neutrophils (Figure 2B-C) and CD4+ T cells (Figure 3B-E) was noticed in CDDP-injured kidneys of Gal-3^-/-^ animals resulting in significant aggravation of renal injury and inflammation.

Gal-3, as a constitutive member of AGE receptor complex, is involved in AGEs removal from circulation and, accordingly, its deficiency results in the aggravation of AGE-induced glomerular injury 27-28. An enhanced AGEs formation in CDDP-injured kidneys and increased activation of AGE receptors on renal DCs promote generation of tolerogenic and immunosuppressive phenotype in renal DCs resulting in DC-mediated attenuation of CDDP-induced AKI 29. Having in mind that aggravated AKI and impaired uptake of AGEs, observed in CDDP-treated Gal-3^-/-^ kidneys (Figure 1A-D) corresponded with reduced immunosuppressive and nephroprotective function of Gal-3^-/-^DCs (Figure 6-7), we assume that Gal-3 deficiency resulted in unstable expression of AGE receptor complex and significantly impeded AGE receptor-dependent generation of tolerogenic phenotype in renal DCs.

Since membranous Gal-3 lacks intracellular peptide sequence with enzymatic activity, it has to associate with other membrane-bound receptors in order to transduce signals from activated AGE receptor 28. CDDP treatment up-regulated expression of Gal-3 in injured kidneys (Figure 1E). An increased expression of Gal-3 was accompanied with enhanced expression of TLR-2 (Figure 1F), while Gal-3 deficiency attenuated CDDP-caused up-regulation of TLR-2 (Figure 1E-F). It is well known that activation of TLR-2 protects against CDDP-induced nephrotoxicity by inducing autophagy which delays apoptosis of PTECs 3. Accordingly, Gal-3 deficiency and reduced expression of TLR-2 increased susceptibility to CDDP-induced apoptosis (Figure 1D), while administration of rGal-3 significantly attenuated CDDP-induced AKI in Gal-3^-/-^ mice by preventing Bax and caspase-3-dependent apoptosis (Figure 2E-H). CDDP induces apoptosis of PTECs through the activation of the intrinsic mitochondrial pathway driven by Bax and caspase-3 61. Gal-3, after exposure to apoptotic stimuli, translocates from the cytosol or nucleus to the mitochondria and attenuates apoptosis by inhibiting Bax oligomerization 62-63. Most of Gal-3 inhibitors (Davanat, modified citrus pectin (MCP), G3-C12) bind to the Gal-3 at the carbohydrate recognition domain (CRD) and prevent Gal-3:Bax interaction 64. Accordingly, significantly increased Bax and caspase-3-dependent apoptosis and aggravated AKI were observed in CDDP+Davanat-treated animals (Figure 2A-D). However, it should be noted that capacity of Gal-3 inhibitors for modulation of CDDP-induced apoptosis probably depends on their selectivity and should be further investigated since opposite, pro- and anti-apoptotic effects were recently observed in MCP-treated cells 25, 63.

While Harazono and colleagues demonstrated that MCP suppressed Gal:Bax heterodimerization and promoted apoptosis 63, Li and co-workers showed that MCP attenuated CDDP-induced AKI by suppressing protein kinase C-α (PKC-α)-driven apoptosis 25. These opposite findings may be explained by the fact that MCP, in similar manner as PKC-α, interferes with multiple intracellular pathways and, therefore, could acts as pro- or anti-apoptotic agent, depending upon cell type or tissue microenvironment 65-68. Furthermore, different effects of Gal-3 inhibition in modulation of CDDP-induced apoptosis could be due to the different doses of CDDP (16mg/kg vs. 20mgkg body weight) which were used in our and in study conducted by Li and colleagues 25. There is evidence that different doses of CDDP may differentially affect development and progression of AKI in mice and in some cases may even hamper the comparison and interpretation of the results 69.

Several lines of evidence demonstrated that Gal-3 associates with TLR-2 on the cell membrane of macrophages and DCs in order to modulate macrophage or DC-driven immune response 52-53. The extent of AKI was not different between CDDP-treated WT recipients of TLR-2-primed WT and Gal-3^-/-^macrophages (Figure S1), suggesting that genetic deletion of Gal-3 did not affect immunomodulatory capacity of TLR-2-activated macrophages. On the contrary, strong positive correlation between Gal-3 and TLR-2-expressing renal DCs (Figure 7A), high percentage of Gal-3-expressing cells in the population of TLR-2-expressing renal DCs (Figure 7B) and significantly reduced presence of TLR-2-expressing DCs in injured kidneys of CDDP-treated Gal-3^-/-^mice (Figure 7C) indicated the important role of Gal-3 for TLR-2-dependent activation of renal DCs.

It is well known that expression of TLR-2 is needed for optimal AGE receptor-dependent generation of immunosuppressive phenotype in renal DCs 2-3, 70-71. DAMPs, released from CDDP-injured PTECs, bind to TLR-2 which interacts with activated AGE receptors to induce development of immature, tolerogenic phenotype in DCs 2, 70-72. In line with these findings, activation of TLR-2 down-regulates expression of co-stimulatory (CD80 and CD86) and MHC-class II molecules on DCs, reduces production of Th1 (IL-12) and Th17-related (IL-1, IL-6, IL-23, TGF-β) cytokines and attenuates capacity of DCs to induce differentiation of naïve CD4+ T cells in effector IFN-γ-producing Th1 and IL-17-producing Th17 cells 2, 70-72. In line with these findings, significantly reduced number of TLR-2 expressing DCs, observed in CDDP-injured kidneys of Gal-3^-/-^ mice (Figure 7C), corresponded to the higher number of CD80, CD86 and MHC class II-expressing (Figure 6D-F) and IL-12 or IL23-producing DCs (Figure 6G-H). Accordingly, significantly elevated serum levels of Th1 and Th17-related cytokines (Figure 1H), and remarkably higher number of renal-infiltrated IFN-γ-producing, T-bet-expressing Th1 cells (Figure 4B-C) and IL-17-producing, RORγT-expressing Th17 cells (Figure 4D-E) were noticed in CDDP-treated Gal-3^-/-^ mice, suggesting the important role of Gal-3 for TLR-2-dependent modulation of DC:T cell cross-talk in CDDP-injured kidneys.

It is well known that activation of TLR-2 promotes production of anti-inflammatory IL-10 in DCs 73 which plays an important role in DC-dependent attenuation of CDDP-induced AKI 5. In line with these findings, significantly lower number of TLR-2 expressing DCs, observed in the kidneys of CDDP-treated Gal-3 deficient mice (Figure 7B), was accompanied by significantly lower serum levels of IL-10 (Figure 1I) and notably reduced number of IL-10-producing renal DCs (Figure 6B), suggesting that Gal-3 was necessary for optimal TLR-2 induced production of immunosuppressive IL-10 in renal DCs. Accordingly, adoptive transfer of WTDCs^Pam3CSK4^ significantly increased serum levels of IL-10 (Figure 8E and Figure 9E) and efficiently attenuated AKI in CDDP-treated WT and Gal-3^-/-^ mice (Figure 8A-D and Figure 9A-D). This phenomenon was not observed in CDDP-injured WT and Gal-3^-/-^ recipients that received Gal-3^-/-^DCs^Pam3CSK4^ (Figure 8-9), indicating crucial importance of Gal-3 for optimal TLR-2-induced production of anti-inflammatory IL-10 in renal DCs.

In addition to IL-10, TLR-2-priming also triggers expression of immunosuppressive IDO1 resulting in the generation of tolerogenic phenotype in DCs 74. In line with these findings, intracellular staining revealed that most of Gal-3+TLR-2+ renal DCs expressed immunosuppressive IDO1 (Figure 7C) and that majority of TLR-2+Gal-3+IDO1+ renal DCs were anti-inflammatory, IL-10-producing cells (Figure 7F), suggesting that Gal-3:TLR-2 cross-talk was involved in generation of immunosuppressive and tolerogenic phenotype in renal DCs. TLR-2 priming results in increased activation of Phosphoinositide 3-kinase (PI3K)/Akt signaling pathway in DCs leading to the enhanced production of IL-10 75. Activated TLR-2 recruits PI3K which converts phosphatidylinositol 4,5-bisphosphate (PIP2) to phosphatidylinositol 3,4,5-trisphosphate (PIP3). PIP3 enables activation of Akt. Activated Akt, on turn, inactivates Glycogen Synthase Kinase 3 (GSK3) and promotes nuclear accumulation of cAMP Response Element-Binding Protein (CREB) which displaces NF-κB p65 from the co-activator of transcription (CREB binding protein (CBP)). Accordingly, enhanced transcriptional activity of CREB leads to the reduced transcriptional activity of NF-κB p65 and results in increased IL-10 production and decreased production of pro-inflammatory cytokines in TLR-2-primed DCs 75. Gal-3 stimulates activation of PI3K/Akt signaling pathway in macrophages and promotes their conversion in M2 immunosuppressive phenotype in PI3K/Akt-dependent manner 76. Although we did formally prove TLR-2:Gal-3 interaction, it is clear that TLR-2 and Gal-3 play in concert. Accordingly, we assume that Gal-3:TLR-2-dependent induction of tolerogenic phenotype in renal DCs was a consequence of increased PI3K/Akt activity.

TLR-2-induced increased expression of IDO1 results in increased production of immunosuppressive KYN in bone-marrow derived DCs 8, 73, 77. In line with these findings, we noticed that activation of TLR-2 significantly enhanced production of KYN in renal DCs (Figure 7E). Importantly, Gal-3 was crucially important for optimal TLR-2-dependent activation of IDO1/KYN pathway in renal DCs. Genetic deletion or pharmacological inhibition of Gal-3 remarkably reduced capacity of renal DCs to secrete KYN upon TLR-2 activation (Figure 7E)*.* Significantly lower number of TLR-2 expressing DCs, found in the kidneys of CDDP-treated Gal-3 deficient mice (Figure 7C), was accompanied by significantly lower serum levels of KYN (Figure 1J) and notably reduced number of IDO1-expressing DCs in CDDP-injured kidneys (Figure 6C).

DCs, in IDO1/KYN-dependent manner, promotes activation of Treg lineage-defining transcription factor (forkhead box P3-FoxP3) in CD4+T cells enabling generation of immunosuppressive CD4+FoxP3+Tregs in inflamed and injured tissues 8. In line with these findings, we showed that Gal-3 and IDO1 are members of the same TLR-2-initiated signaling pathway which was responsible for DCs:Tregs cross-talk in CDDP-injured kidneys. Gal-3-dependent nephroprotective effects of TLR-2-activated renal DCs has been relied on the IDO1-induced expansion of renal-infiltrating Tregs since either inhibition of IDO1 activity in TLR-2-primed renal DCs (Figure 8-9) or depletion of Tregs (Figure 11) completely diminished DC^Pam3CSK4^-mediated attenuation of CDDP-induced AKI.

Tolerogenic DCs, in IDO1/KYN-dependent manner, prevent transdifferention of resting Tregs in effector Th1 or Th17 cells 77. During TCR-mediated activation, signals via the protein kinase B (PKB/Akt) and mammalian target of rapamycin (mTOR) cause reprogramming of Tregs into inflammatory IFN-γ and IL-17 producing CD4+T cells 77. Tolerogenic DCs, in IDO1/KYN-dependent manner, induce activation of general control nonderepressible 2 (GCN2) kinase which inhibits Akt/mTOR signaling in Tregs preventing their transdifferention in Th1/Th17 cells 77. Expression of Gal-3 was necessary for optimal activation of IDO1/KYN pathway in renal DCs^Pam3CSK4^ (Figure 7E) and for consequent IDO1/KYN-dependent maintenance of immunosuppressive function of Tregs by DCs^Pam3CSK4^ (Figure 7H-J). Decreased expression and production of anti-inflammatory IL-10 and increased expression and production of inflammatory IFN-γ and IL-17 were noticed in Tregs which had been cultured with Gal-3^-/-^DCs^Pam3CSK4^ or WT DC^Pam3CSK4+Davanat^ compared to Tregs that were cultured with WTDCs^Pam3CSK4^ (Figure 7H-J). Accordingly, adoptive transfer of WTDCs^Pam3CSK4^, but not transfer of Gal-3^-/-^DCs^Pam3CSK4^, significantly increased total number of immunosuppressive, IL-10-producing Tregs and remarkably reduced presence of inflammatory, IFN-γ and IL-17-producing CD4+T cells in the kidneys of CDDP-treated WT and Gal-3^-/-^ recipients (Figure 8-9).

Adoptive transfer of Tregs alleviated CDDP-induced nephrotoxicity 9, indicating Tregs-based therapy as a promising approach for amelioration of acute renal failure and inflammation 10. Although Gal-3 is constitutively expressed on Tregs, Gal-3 deficiency did not alter migratory, nephroprotective and immunosuppressive capacity of Tregs (Figure 5). Nevertheless, expression of Gal-3 on TLR-2-primed DCs was crucially responsible for their capacity to enhance nephroprotective and immunosuppressive properties of Tregs (Figure 10). Tregs, activated by WTDCs^Pam3CSK4^ more efficiently attenuated CDDP-induced nephrotoxicity than WTDC^Pam3CSK4^-non-primed Tregs. Importantly, genetic deletion or pharmacological inhibition of Gal-3 completely diminished capacity of WTDCs^Pam3CSK4^ to enhance nephroprotective properties of Tregs, indicating crucially important role of Gal-3 for the crosstalk of TLR-2-primed renal DCs and Tregs in Tregs-based attenuation of CDDP-induced AKI (Figure 9).

It is well known that Tregs may induce production of immunosuppressive cytokines in neutrophils 78 leading to the attenuation of AKI 79. In line with these findings, significantly lower number of renal-infiltrated TLR-2 expressing DCs (Figure 7C) and consequently reduced presence of Tregs (Figure 4G) were accompanied with reduced presence of IL-10-producing neutrophils in injured kidneys of CDDP-treated Gal-3^-/-^ mice (Figure 3E). Additionally, Tregs, which had been cultured with WTDCs^Pam3CSK4^ promoted expression of IL-10 and suppressed expression of inflammatory cytokines (IFN-γ and IL-17) in neutrophils (Figure 7F and Figure 10D-E), while Tregs generated in the presence of Gal-3^-/-^DCs^Pam3CSK4^or WT DCs^Pam3CSK4+Davanat^ were not able to generate immunosuppressive phenotype in activated neutrophils (Figure 7F). Accordingly, in contrast to WTDCs^Pam3CSK4^, adoptive transfer of Gal-3^-/-^DCs^Pam3CSK4^ did not result in expansion of renal-infiltrating Tregs and IL-10 producing neutrophils in the kidneys of CDDP-treated WT and Gal-3^-/-^ recipients (Figure 8-9), indicating the important role of Gal-3 for TLR-2-dependent DC-driven regulation of Tregs:neutrophils crosstalk in injured kidneys.

**Figure 12:**
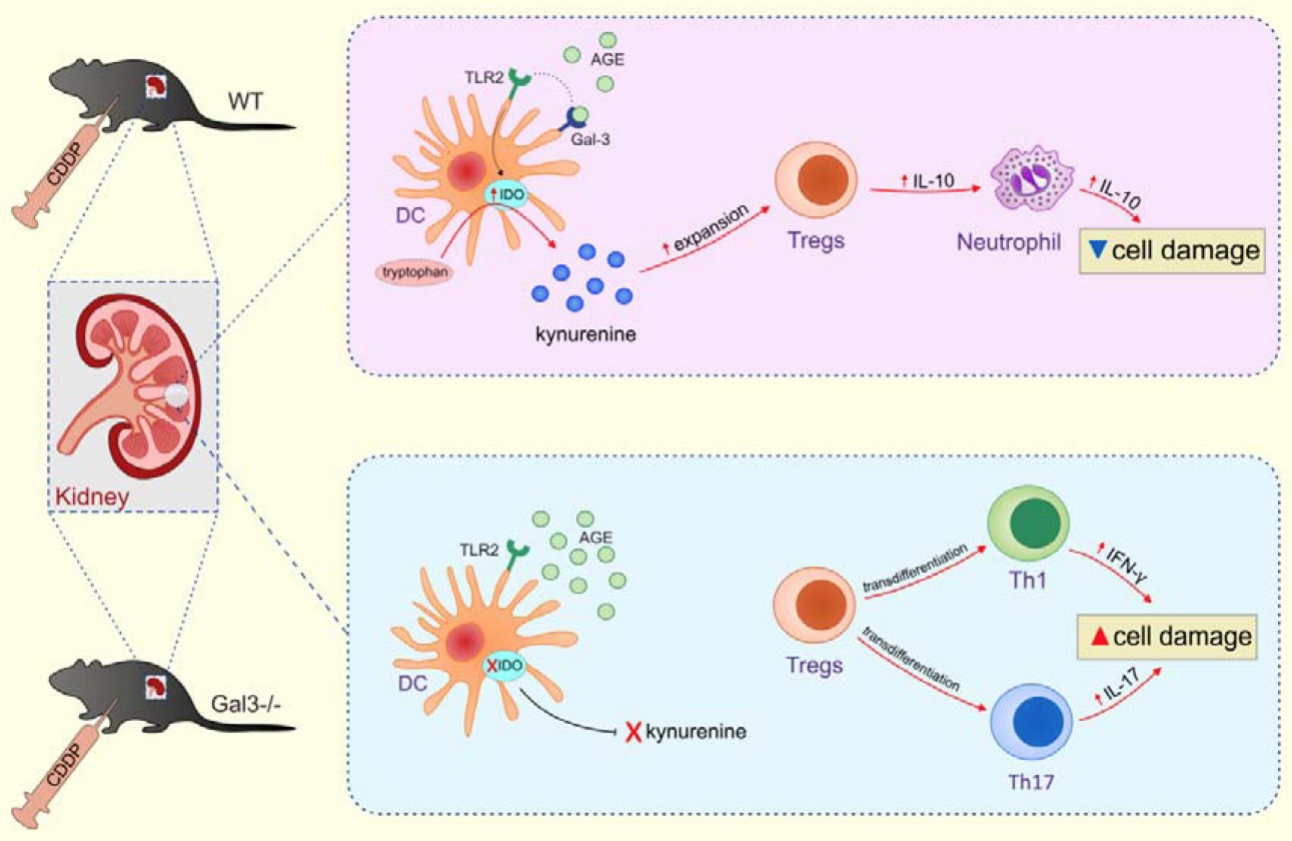
** Expression of Gal-3 in renal DCs is necessary for optimal TLR-2-dependent activation of IDO1/KYN pathway and for consequent expansion of renal-infiltrating Tregs.** Gal-3, expressed on renal DCs, serves as receptor for AGEs which promotes TLR-2-dependent activation of IDO1/KYN pathway and protects against CDDP-caused AKI by inducing expansion of Tregs which, in turn, promote generation of immunosuppressive, IL-10-producing phenotype in renal-infiltrated neutrophils and prevent inflammation driven by IFN-γ- and IL-17-producing neutrophils, Th1 and Th17 cells (upper panel). Gal-3 deficiency resulted in reduced uptake of AGEs, attenuated activation of TLR-2-dependent IDO1/KYN pathway in renal DCs, decreased production of KYN, reduced presence of immunosuppressive Tregs and enhanced expansion of inflammatory IFN-γ- and IL-17-producing neutrophils, Th1 and Th17 cells in the kidneys, resulting in significant aggravation of CDDP-induced AKI (lower panel).

In conclusion, we propose that expression of Gal-3 in renal DCs is crucially important for their protective effects in CDDP-induced AKI. The main mechanism by which Gal-3 regulates immunosuppressive capacity of renal DCs is relied on the TLR-2-dependent activation of IDO1/KYN pathway and consequent expansion of IL-10-producing renal-infiltrated Tregs which suppress neutrophil-, Th1- and Th17 cell-driven inflammation in CDDP-injured kidneys (Figure 12). Activation of Gal-3:TLR-2:IDO1 pathway in renal DCs should be further explored as new therapeutic approach for DC-based immunosuppression of inflammatory renal diseases.

## Supplementary Material

Supplementary figures.

## Abbreviations

AGEs: advanced glycosylation end products
AKI: acute kidney injury
APC: allophycocyanin
CDDP: cisplatin
CY: cyclophosphamide
DAMPs: damage-associated molecular patterns
DCs: dendritic cells
ELISA: enzyme-linked immunosorbent assay
FITC: fluorescein isothiocyanate
FoxP3: forkhead box P3
Gal-3: galectin 3
H&E: hematoxylin and eosin
IDO1: indoleamine 2,3-dioxygenase 1
IFN-γ: interferon gamma
IHC: immunohistochemistry
IL: interleukin
KYN: kynurenine
1-MT: 1-methyl tryptophan
PerCP: peridinin chlorophyll protein
PE: phycoerythrin
PTECs: proximal tubular epithelial cells
ROS: reactive oxygen species
SEM: standard error of the mean
TLR: toll-like receptor
Tregs: T regulatory cells
WT: wild type
