# Supplementary material for: Galectin 3 protects from cisplatin-induced acute kidney injury by promoting TLR-2-dependent activation of IDO1/Kynurenine pathway in renal DCs
Source: Theranostics. 2019 Aug 14;9(20):5976–6001. doi: 10.7150/thno.33959 (PMC6735380; doi:10.7150/thno.33959)

Galectin 3 protects from cisplatin-induced acute kidney injury by promoting TLR-2-dependent activation of IDO1/Kynurenine pathway in renal DCs

Vladislav Volarevic[1*], Bojana Simovic Markovic[1], Marina Gazdic Jankovic[2], Bojana Djokovic[1], Nemanja Jovicic[3], C. Randall Harrell[4], Crissy Fellabaum[4], Valentin Djonov[5], Nebojsa Arsenijevic[1], and Miodrag L. Lukic[1]

[1]Department of Microbiology and Immunology, Center for Molecular Medicine and Stem Cell Research, Faculty of Medical Sciences, University of Kragujevac, 69 Svetozar Markovic Street, Kragujevac, Serbia

[2]Department of Genetics, Faculty of Medical Sciences, University of Kragujevac, 69 Svetozar Markovic Street, Kragujevac, Serbia

[3]Department of Histology and Embryology, Faculty of Medical Sciences, University of Kragujevac, 69 Svetozar Markovic Street, Kragujevac, Serbia

[4]Regenerative Processing Plant, LLC, 34176 US Highway 19 N Palm Harbor, Palm Harbor, Florida, United States of America

[5]Institute of Anatomy, University of Bern, 2 Baltzerstrasse, Switzerland

*Corresponding author: Prof. dr. Vladislav Volarevic

Postal address: 69 Svetozar Markovic Street, 34000 Kragujevac, Serbia

e-mail: drvolarevic@yahoo.com

telephone number/fax number: +38134306800

Supplementary figure legends

Supplementary Figure 1. Davanat significantly attenuated expression of Gal-3 in the kidneys. Davanat was intraperitoneally injected in CDDP-treated WT animals (100 μg/day), for three consecutive days before CDDP administration (16 mg/kg body weight). Representative immunohistochemical images of renal tissue sections showing expression of Gal-3 in the cortex of saline-only, saline+Davanat, CDDP-only and CDDP+Davanat-treated kidneys (left panel). Bar graphs showing significantly attenuated expression of Gal-3 in the kidneys of saline+Davanat ($p<0.01$) and CDDP+Davanat-treated mice ($p<0.001$) compared to saline-only and CDDP-only-treated animals, respectively (right panel). Values are Mean ± standard error of the mean (SEM) (n=6 mice per group). **$p<0.001$, ***$p<0.0001$.

Supplementary Figure 2. Gal-3 deletion did not affect nephroprotective and immunosuppressive capacity of TLR-2-primed macrophages in CDDP-induced AKI. Macrophages, isolated from healthy WT and Gal-3$^{-/-}$ mice, were stimulated with Pam3CSK4 (300 ng/mL; WTM$\phi^{Pam3CSK4}$ and Gal-3$^{-/-}$M$\phi^{Pam3CSK4}$) and injected via the tail vein ($1x10^6$/ mouse) 2 h before administration of CDDP (16 mg/kg body weight). There was no significant difference in serum levels of urea and creatinine (A), histological score (B) and the extent of renal injury, observed in cortex of CDDP-treated kidneys of mice that received WTM$\phi^{Pam3CSK4}$ and Gal-3$^{-/-}$M$\phi^{Pam3CSK4}$. Data from two individual experiments with 8 mice per group are shown as Mean ± SEM.

Supplementary Figure 3. Representative density plots showing neutrophils and CD4+ T cells which infiltrated kidneys of CDDP-treated WT mice that received WT Tregs and Gal-3$^{-/-}$Tregs. WT Tregs and Gal-3$^{-/-}$Tregs ($1x10^6$ Tregs/ mouse) were intravenously injected in

CDDP-treated WT animals 18 h before induction of AKI. Representative density plots showing IFN-γ and IL-17-producing cells gated in the population of Gr-1+CD45+ neutrophils (A) and IFN-γ, IL-17-producing and FoxP3-expressing cells gated in the population of CD4+ T cells (B).

Supplementary Figure 4. Representative density plots showing Tregs before and after culture with WTDCs^{Pam3CSK4}, WTDC$^{Pam3CSK4+Davanat}$ or Gal-3$^{-/-}$DCs^{Pam3CSK4}. Tregs and DCs were cultured physically separated using a 0.4 µm porous transwell system. After 48 h of culture, non-primed Tregs or Tregs primed with WTDCs^{Pam3CSK4}, WTDC$^{Pam3CSK4+Davanat}$ or Gal-3$^{-/-}$DCs^{Pam3CSK} were collected and used for intracellular staining and flow cytometry analysis. IFN-γ, IL-17, and IL-10-producing cells gated in the population of FoxP3-expressing cells which were previously gated in the population of CD4+CD25+cells.

Supplementary Figure 5. Transfer of WTDCs^{Pam3CSK4} significantly attenuated CDDP-induced AKI in WT recipients by altering cytokine production in renal-infiltrated CD4+T cells and neutrophils. TLR-2-primed DCs, isolated from the kidneys of untreated WT and Gal-3$^{-/-}$ mice (WTDCs^{Pam3CSK4} and Gal-3$^{-/-}$DCs^{Pam3CSK4}), were intravenously injected (5×10^{5}cells/ mouse) in CDDP-treated WT recipients (WT^{WTDCsPam3CSK4} and WT$^{Gal_3-/-DCsPam3CSK4}$) two days prior CDDP administration (16 mg/kg body weight). IDO1 was inhibited in TLR-2-primed renal DCs (WTDCs$^{Pam3CSK4+1-MT}$) and Gal-3$^{-/-}$DCs (Gal-3$^{-/-}$DCs$^{Pam3CSK4+1-MT}$) by using 1-methyl tryptophan (1-MT; 2 mM). Representative density plots showing IL-10-producing, FoxP3-expressing, IL-17 and IFN-γ-producing cells gated in the population of CD4+T cells (A) and IL-10-, IL-17- and IFN-γ-producing cells gated in the population of CD45+Gr-1+ neutrophils (B).

Supplementary Figure 6. **Transfer of WTDCs^{Pam3CSK4} significantly attenuated CDDP-induced AKI in Gal-3$^{-/-}$ recipients by altering cytokine production in renal-infiltrated CD4+T cells and neutrophils.** TLR-2-primed DCs, isolated from the kidneys of untreated WT

and Gal-3$^{-/-}$ mice (WTDCs^{Pam3CSK4} and Gal-3$^{-/-}$DCs^{Pam3CSK4}), were intravenously injected (5×10^{5}cells/ mouse) in CDDP-treated Gal-3$^{-/-}$ recipients (Gal-3$^{-/-WTDCsPam3CSK4}$ and Gal-3$^{-/-Gal_3-/-DCsPam3CSK4}$) two days prior CDDP administration (16 mg/kg body weight). IDO1 was inhibited in TLR-2-primed renal DCs (WTDCs$^{Pam3CSK4+1-MT}$) and Gal-3$^{-/-}$DCs (Gal-3$^{-/-}$DCs$^{Pam3CSK4+1-MT}$) by using 1-methyl tryptophan (1-MT; 2 mM). Representative density plots showing IFN-γ-, IL-17-, IL-10-producing cells gated in the population of CD45+Gr-1+ neutrophils (A) and IL-17-,IFN-γ-producing, FoxP3-expressing and IL-10-producing cells gated in the population of CD4+T cells (B).

Supplementary Figure 7. Expression of Gal-3 on TLR-2-primed DCs is crucially important for their capacity to enhance Tregs-based modulation of cytokine production in renal-infiltrated neutrophils. For the depletion of Tregs, anti-CD25 monoclonal antibody (250 μg/mouse) was intraperitoneally given to mice 3 days before CDDP administration (16 mg/kg body weight). For transfer experiments non-primed Tregs, Tregs primed with WTDC^{Pam3CSK4}, WTDC$^{Pam3CSK4+Davanat}$ or Gal-3$^{-/-}$ DC^{Pam3CSK4} (1x10^{6}Tregs/ mouse) were intravenously injected in CDDP-treated animals 18 h before induction of AKI. Representative density plots showing T-bet-expressing, IFN-γ-, IL-17-, TNF-α-, IL-10-producing cells gated in the population of CD45+Gr-1+ neutrophils.

Supplementary Figure 8. Depletion of Tregs completely diminished Gal-3-dependent capacity of TLR-primed renal DCs to suppress production of inflammatory cytokines in neutrophils and CD4+T cells of CDDP-injured kidneys. For the depletion of Tregs, CDDP-treated WT^{WTDCPam3CSK4} and Gal-3$^{-/-WTDCPam3CSK4}$ mice received either cyclophosphamide (CY; 10 mg/kg) 3 days before CDDP administration (16 mg/kg body weight) or anti-CD25 (P61) monoclonal antibody (250 μg per mouse). Representative density plots showing IFN-γ- and IL-

17-producing cells gated in the population of CD45+Gr-1+ neutrophils (A) and IFN-γ-, IL-17-producing and FoxP3-expressing cells gated in the population of CD4+T cells (B).

Supplementary Figure 1

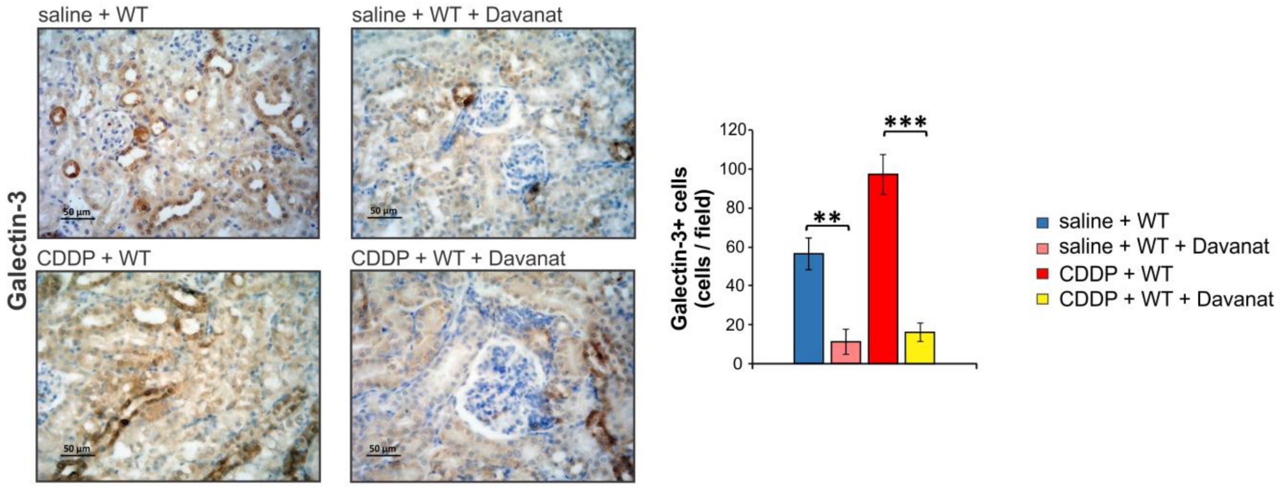

Supplementary Figure 2

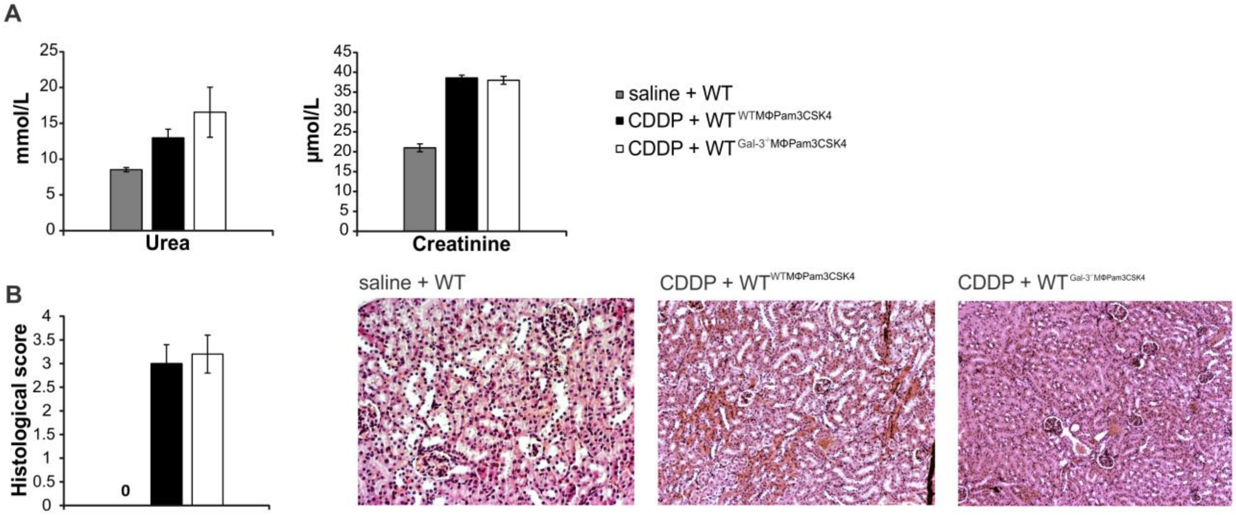

Supplementary Figure 3

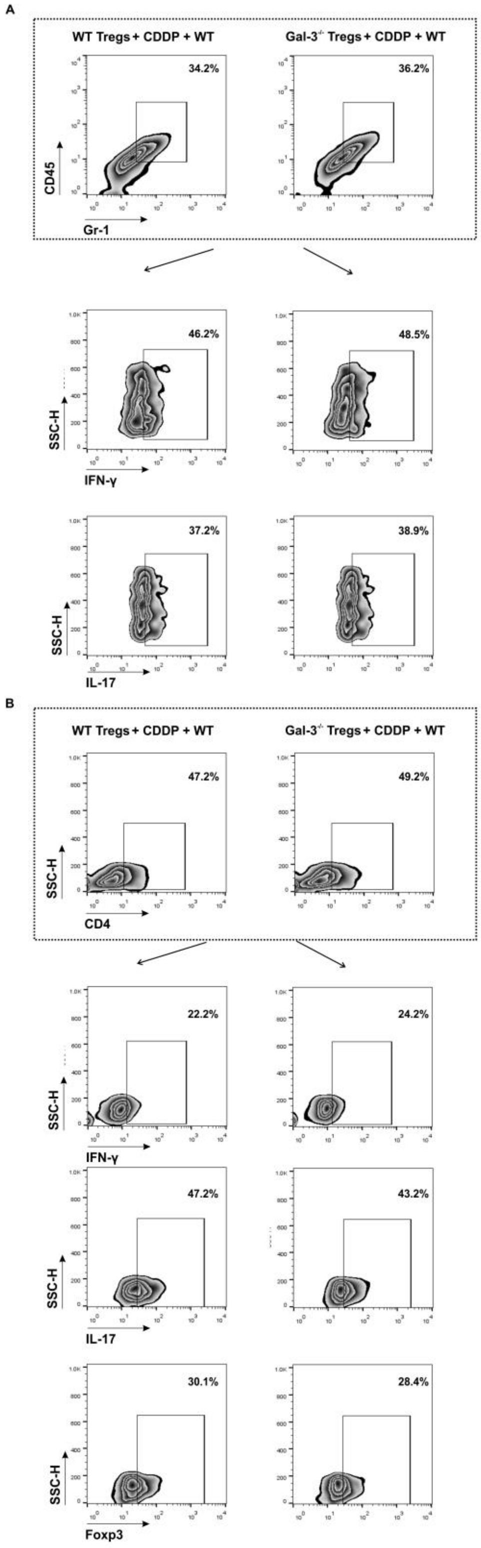

Supplementary Figure 4

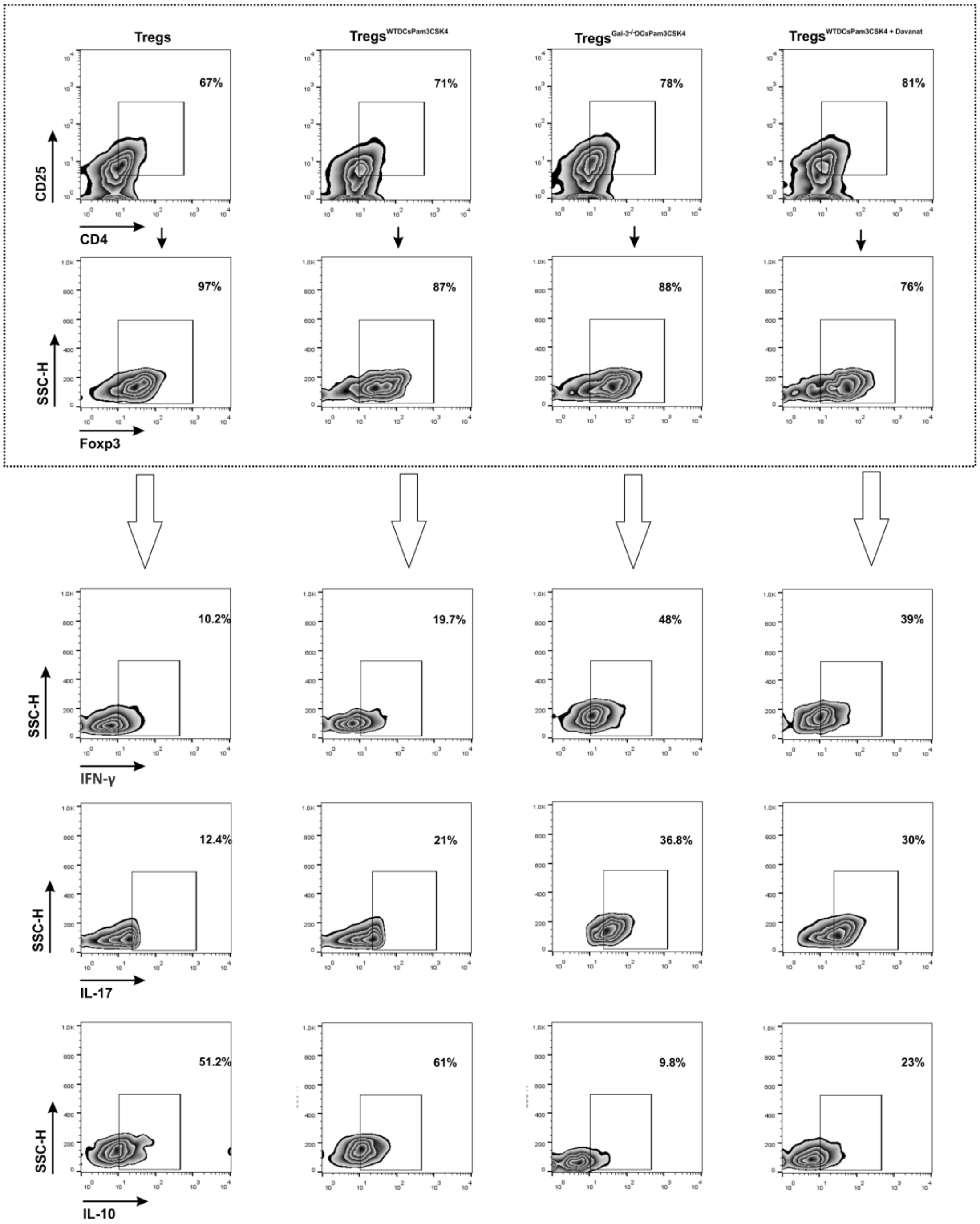

Supplementary Figure 5

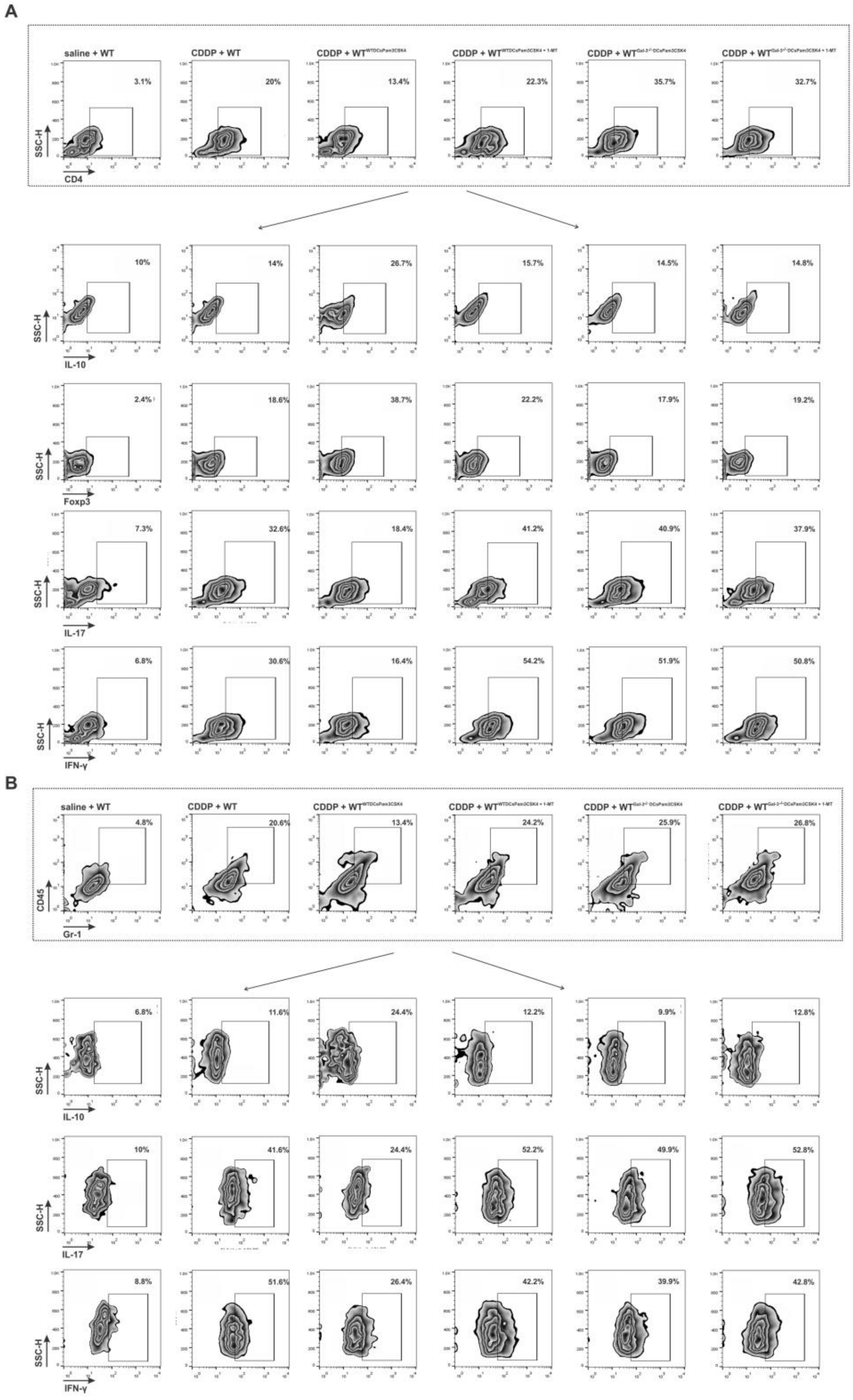

Supplementary Figure 6

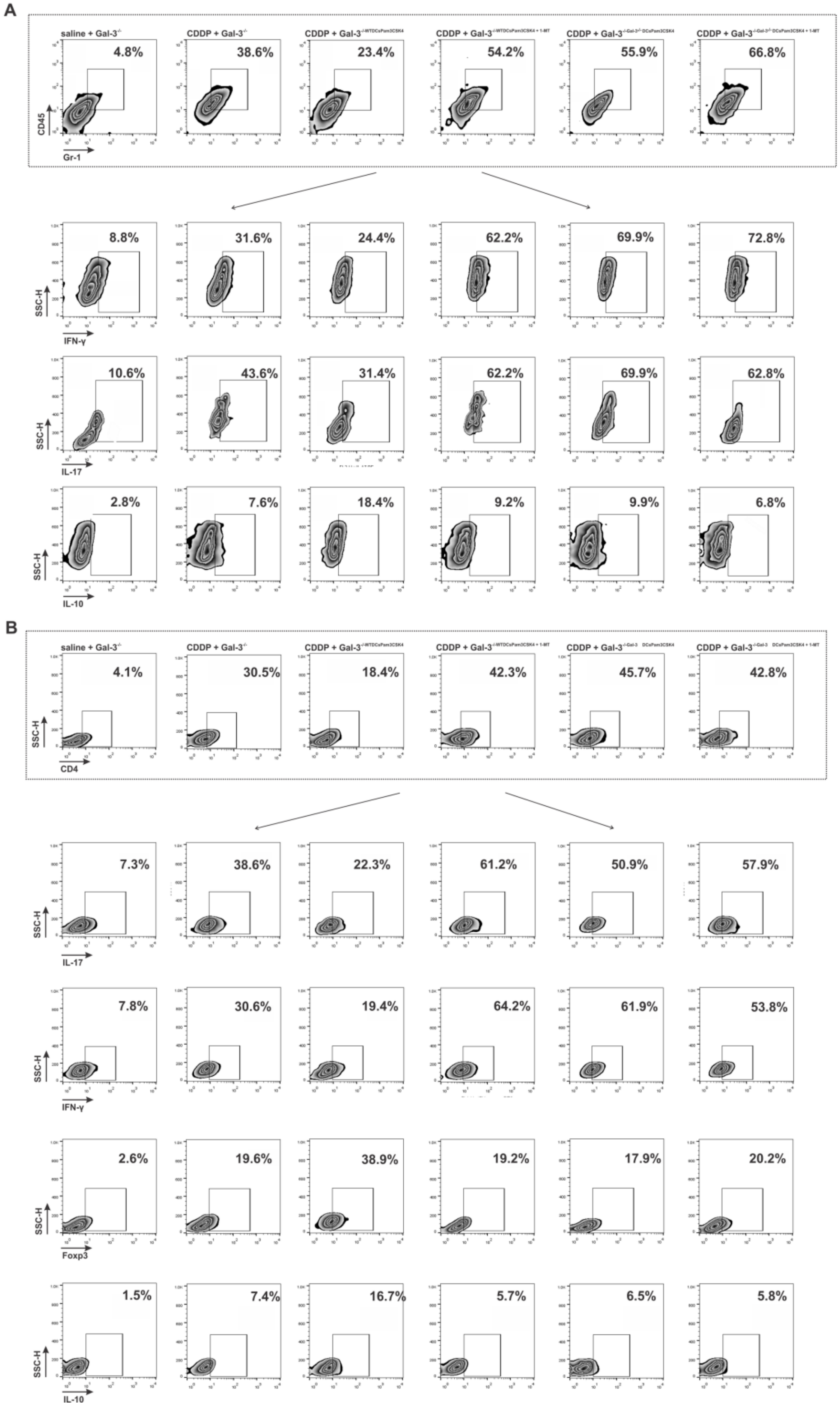

Supplementary Figure 7

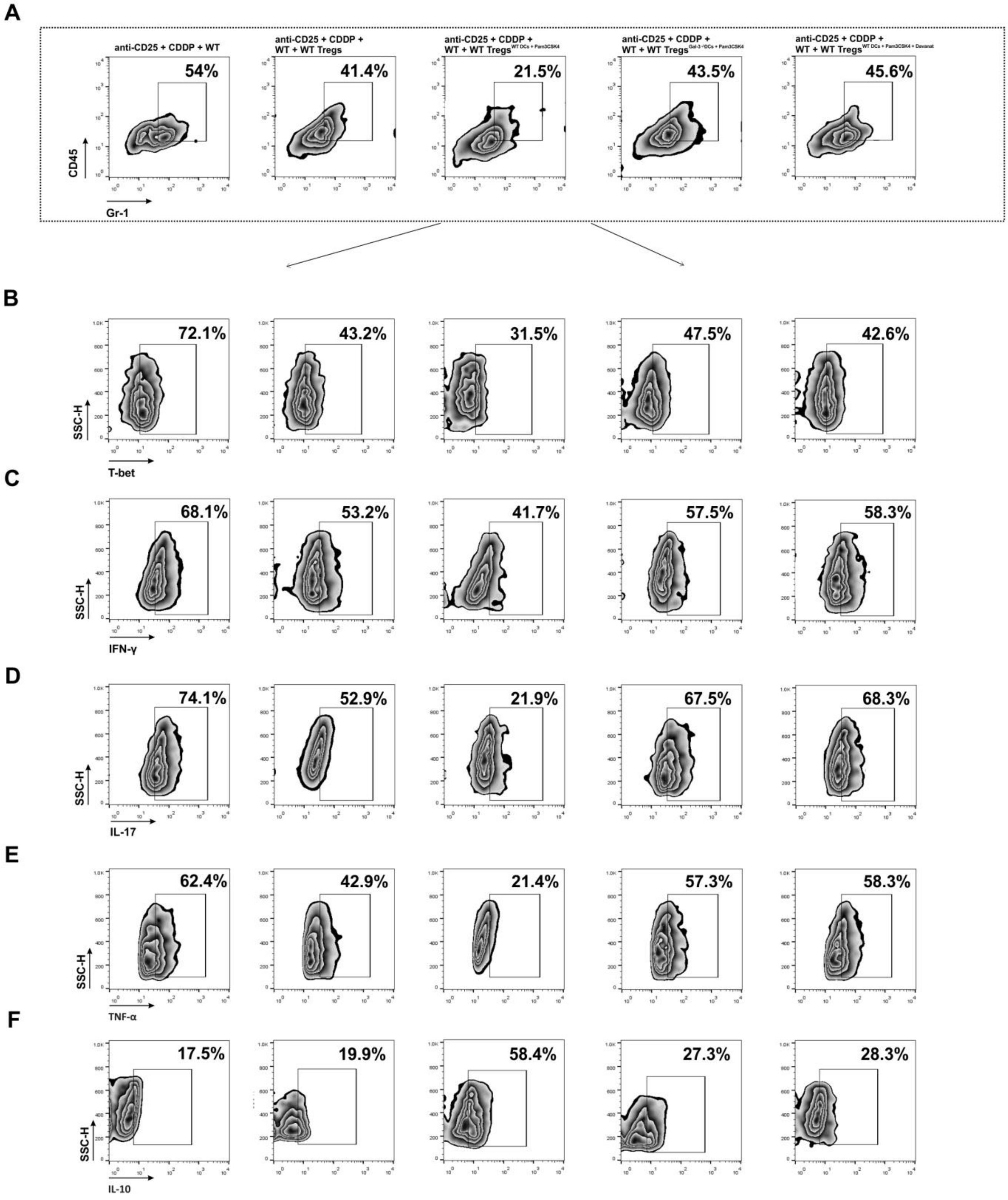

Supplementary Figure 8

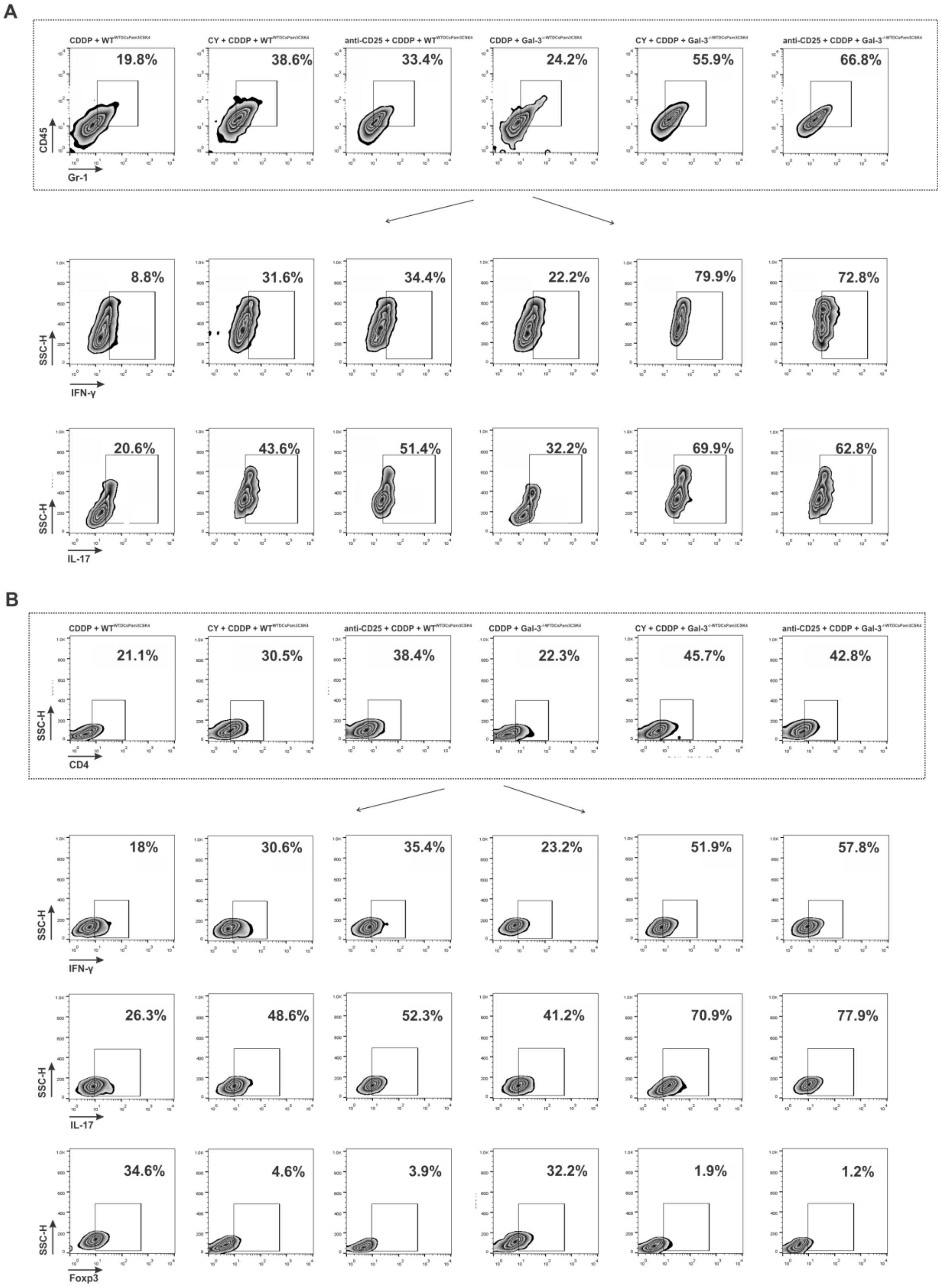

Supplement: Supplementary file 1 — Supplementary figures. [file thnov09p5976s1.pdf]
